# Supplementary material for: Umbrella review of international evidence for the effectiveness of school-based physical activity interventions
Source: PLoS One. 2024 Jun 13;19(6):e0304513. doi: 10.1371/journal.pone.0304513 (PMC11175402; doi:10.1371/journal.pone.0304513)
Supplement: S1 File — (DOCX) [file pone.0304513.s002.docx]

**S1 Appendix: The Search Strategy**

(Physical inactivity OR physical activity OR sedentary lifestyle OR school-based intervention OR school-based program OR school-based) AND (health policy OR polic* OR health promotion OR health impact OR school*) AND (young OR adolescent OR youth OR children OR teenager) AND (systematic OR Review OR meta-analysis).

**S2 Appendix: Studies were excluded during full-text screening with reasons.**

1. **Inappropriate target population (n=8)**

Manojlovic, M., Roklicer, R., Trivic, T., Milic, R., Maksimović, N., Tabakov, R., Sekulic, D., Bianco, A. and Drid, P., 2023. Effects of school-based physical activity interventions on physical fitness and cardiometabolic health in children and adolescents with disabilities: a systematic review. *Frontiers in Physiology*, *14*, p.1180639.

Craike, M., Wiesner, G., Hilland, T.A. and Bengoechea, E.G., 2018. Interventions to improve physical activity among socioeconomically disadvantaged groups: an umbrella review. *International Journal of Behavioral Nutrition and Physical Activity*, *15*(1), pp.1-11.

Ho, T.J.H., Cheng, L.J. and Lau, Y., 2021. School-based interventions for the treatment of childhood obesity: a systematic review, meta-analysis and meta-regression of cluster randomised controlled trials. *Public Health Nutrition*, *24*(10), pp.3087-3099.

Bleich, S.N., Vercammen, K.A., Zatz, L.Y., Frelier, J.M., Ebbeling, C.B. and Peeters, A., 2018. Interventions to prevent global childhood overweight and obesity: a systematic review. *The lancet Diabetes & endocrinology*, *6*(4), pp.332-346.

Eddy, L.H., Wood, M.L., Shire, K.A., Bingham, D.D., Bonnick, E., Creaser, A., Mon‐Williams, M. and Hill, L.J., 2019. A systematic review of randomized and case‐controlled trials investigating the effectiveness of school‐based motor skill interventions in 3‐to 12‐year‐old children. *Child: care, health and development*, *45*(6), pp.773-790.

Hodder, R.K., O'Brien, K.M., Lorien, S., Wolfenden, L., Moore, T.H., Hall, A., Yoong, S.L. and Summerbell, C., 2022. Interventions to prevent obesity in school-aged children 6-18 years: An update of a Cochrane systematic review and meta-analysis including studies from 2015–2021. *EClinicalMedicine*, *54*.

Mijalković, S., Stanković, D., Tomljanović, M., Batez, M., Grle, M., Grle, I., Brkljačić, I., Jularić, J., Sporiš, G. and Fišer, S.Ž., 2022. School-Based Exercise Programs for Promoting Cardiorespiratory Fitness in Overweight and Obese Children Aged 6 to 10. *Children*, *9*(9), p.1323.

Fenesi, B., Graham, J.D., Crichton, M., Ogrodnik, M. and Skinner, J., 2022. Physical activity in high school classrooms: A promising avenue for future research. *International Journal of Environmental Research and Public Health*, *19*(2), p.688.

1. **Being ongoing studies (n=2)**

Demetriou, Y., Reimers, A.K., Alesi, M., Scifo, L., Borrego, C.C., Monteiro, D. and Kelso, A., 2019. Effects of school-based interventions on motivation towards physical activity in children and adolescents: protocol for a systematic review. *Systematic reviews*, *8*, pp.1-6.

Campos-Garzón, P., Sevil-Serrano, J., Barranco-Ruíz, Y. and Chillón, P., 2020. Objective measures to assess active commuting physical activity to school in young people: a systematic review protocol and practical considerations. *International journal of environmental research and public health*, *17*(16), p.5936.

1. **Being not school-based (n=5)**

Cassar, S., Salmon, J., Timperio, A., Naylor, P.J., Van Nassau, F., Contardo Ayala, A.M. and Koorts, H., 2019. Adoption, implementation and sustainability of school-based physical activity and sedentary behaviour interventions in real-world settings: a systematic review. *International Journal of Behavioral Nutrition and Physical Activity*, *16*, pp.1-13.

Kokenge, M.C., Ruppar, T.M. and Buchholz, S., 2022. Physical Activity Interventions Among American Indian and Alaska Native Persons: A Systematic Review. *American Journal of Health Promotion*, *36*(8), pp.1350-1370.

Abu-Omar, K., Rütten, A., Burlacu, I., Schätzlein, V., Messing, S. and Suhrcke, M., 2017. The cost-effectiveness of physical activity interventions: a systematic review of reviews. *Preventive medicine reports*, *8*, pp.72-78.

Nash, E.A., Critchley, J.A., Pearson, F., Awad, S.F., Abu-Raddad, L.J., Abu-Hijleh, F.M. and Huangfu, P., 2021. A systematic review of interventions to promote physical activity in six Gulf countries. *PLoS One*, *16*(10), p.e0259058.

Lane, C., McCrabb, S., Nathan, N., Naylor, P.J., Bauman, A., Milat, A., Lum, M., Sutherland, R., Byaruhanga, J. and Wolfenden, L., 2021. How effective are physical activity interventions when they are scaled-up: a systematic review. *International Journal of Behavioral Nutrition and Physical Activity*, *18*(1), pp.1-11.

1. **Measured different outcomes rather than PA (n=17)**

Wu, J., Yang, Y., Yu, H., Li, L., Chen, Y. and Sun, Y., 2023. Comparative effectiveness of school-based exercise interventions on physical fitness in children and adolescents: a systematic review and network meta-analysis. *Frontiers in Public Health*, *11*.

Saavedra Dias, R., Barros, A.N., Silva, A.J., Leitão, J.C., Narciso, J., Costa, A.M. and Tallon, J.M., 2020. The effect of school intervention programs on the body mass index of adolescents: a systematic review with meta-analysis. *Health Education Research*, *35*(5), pp.396-406.

Pulling Kuhn, A., Stoepker, P., Dauenhauer, B. and Carson, R.L., 2021. A systematic review of multi-component comprehensive school physical activity program (CSPAP) interventions. *American Journal of Health Promotion*, *35*(8), pp.1129-1149.

Verjans-Janssen, S.R., van de Kolk, I., Van Kann, D.H., Kremers, S.P. and Gerards, S.M., 2018. Effectiveness of school-based physical activity and nutrition interventions with direct parental involvement on children’s BMI and energy balance-related behaviors–A systematic review. *PloS one*, *13*(9), p.e0204560.

Podnar, H., Jurić, P., Karuc, J., Saez, M., Barceló, M.A., Radman, I., Starc, G., Jurak, G., Đurić, S., Potočnik, Ž.L. and Sorić, M., 2021. Comparative effectiveness of school‐based interventions targeting physical activity, physical fitness or sedentary behaviour on obesity prevention in 6‐to 12‐year‐old children: A systematic review and meta‐analysis. *Obesity Reviews*, *22*(2), p.e13160.

Arlinghaus, K.R., Cepni, A.B., Helbing, R.R., Goodman, L.P., Ledoux, T.A. and Johnston, C.A., 2022. Response to school‐based interventions for overweight and obesity: A systematic scoping review. *Clinical obesity*, *12*(6), p.e12557.

Smit, M.S., Boelens, M., Mölenberg, F.J., Raat, H. and Jansen, W., 2023. The long‐term effects of primary school‐based obesity prevention interventions in children: A systematic review and meta‐analysis. *Pediatric obesity*, *18*(3), p.e12997.

Barbosa, A., Whiting, S., Simmonds, P., Scotini Moreno, R., Mendes, R. and Breda, J., 2020. Physical activity and academic achievement: an umbrella review. *International Journal of Environmental Research and Public Health*, *17*(16), p.5972.

van Loon, A.W., Creemers, H.E., Okorn, A., Vogelaar, S., Miers, A.C., Saab, N., Westenberg, P.M. and Asscher, J.J., 2022. The effects of school‐based interventions on physiological stress in adolescents: A meta‐analysis. *Stress and health*, *38*(2), pp.187-209.

Cerrato-Carretero, P., Roncero-Martin, R., Pedrera-Zamorano, J.D., Lopez-Espuela, F., Puerto-Parejo, L.M., Sanchez-Fernandez, A., Canal-Macias, M.L., Moran, J.M. and Lavado-Garcia, J.M., 2021, April. Long-term dietary and physical activity interventions in the school setting and their effects on BMI in children aged 6–12 years: Meta-analysis of randomized controlled clinical trials. In *Healthcare* (Vol. 9, No. 4, p. 396). MDPI.

Nguyen, V.H., 2018. School-based exercise interventions effectively increase bone mineralization in children and adolescents. *Osteoporosis and Sarcopenia*, *4*(2), pp.39-46.

De Greeff, J.W., Bosker, R.J., Oosterlaan, J., Visscher, C. and Hartman, E., 2018. Effects of physical activity on executive functions, attention and academic performance in preadolescent children: a meta-analysis. *Journal of science and medicine in sport*, *21*(5), pp.501-507.

Buttazzoni, A.N., Van Kesteren, E.S., Shah, T.I. and Gilliland, J.A., 2018. Active school travel intervention methodologies in North America: a systematic review. *American journal of preventive medicine*, *55*(1), pp.115-124.

Taylor, S.L., Noonan, R.J., Knowles, Z.R., Owen, M.B. and Fairclough, S.J., 2018. Process evaluation of a pilot multi-component physical activity intervention–active schools: Skelmersdale. *BMC Public Health*, *18*(1), pp.1-16.

Bradshaw, M., Gericke, H., Coetzee, B.J., Stallard, P., Human, S. and Loades, M., 2021. Universal school-based mental health programmes in low-and middle-income countries: a systematic review and narrative synthesis. *Preventive Medicine*, *143*, p.106317.

Xu, T., Tomokawa, S., Gregorio Jr, E.R., Mannava, P., Nagai, M. and Sobel, H., 2020. School-based interventions to promote adolescent health: A systematic review in low-and middle-income countries of WHO Western Pacific Region. *PloS one*, *15*(3), p.e0230046.

Andermo, S., Hallgren, M., Nguyen, T.T.D., Jonsson, S., Petersen, S., Friberg, M., Romqvist, A., Stubbs, B. and Elinder, L.S., 2020. School-related physical activity interventions and mental health among children: a systematic review and meta-analysis. *Sports medicine-open*, *6*(1), pp.1-27.

1. **Did not adopt a systematic review approach (n=3)**

Lim, C.S., Robinson, J., Hinton, E., Gordy, X.Z., Gamble, A., Compretta, C., Holmes, M.E. and Ravola, M., 2022. School-based obesity prevention programs in rural communities: a scoping review. *JBI evidence synthesis*, *20*(12), p.2936.

Bandeira, A.D.S., Ravagnani, F.C.D.P., Barbosa Filho, V.C., de Oliveira, V.J.M., de Camargo, E.M., Tenório, M.C.M., Sandreschi, P.F., Dos Santos, P.C., Ramires, V.V., Hallal, P.C. and Silva, K.S., 2022. Mapping recommended strategies to promote active and healthy lifestyles through physical education classes: a scoping review. *International Journal of Behavioral Nutrition and Physical Activity*, *19*(1), pp.1-20.

Drouka, A., Brikou, D., Causeret, C., Al Ali Al Malla, N., Sibalo, S., Ávila, C., Alcat, G., Kapetanakou, A.E., Gurviez, P., Fellah-Dehiri, N. and Masson, M., 2023. Effectiveness of school-based interventions in Europe for promoting healthy lifestyle behaviors in children. *Children*, *10*(10), p.1676.

**S3 Appendix: Characteristics of included reviews.**

| **Author, Year** | **Title of the Review** | **The study designs included in the review** | **Studies included number** | **Interventions description** | **Participants type and Age** | **Primary outcomes** | **Interventions Length included in the review** | **Summary of results** |
| --- | --- | --- | --- | --- | --- | --- | --- | --- |
|  |  |  |  |  |  |  |  |  |
| Yuksel et al., 2020  [28] | School-Based Intervention Programs for Preventing Obesity and Promoting Physical Activity and Fitness: A Systematic Review | c-RCTs,  non-RCTs  quasi-experimental  RCTs,  one-group repeated measures designs and mixed-effects models | 19 | PA interventions in the form of increasing the duration of PE or changing its content, performing extracurricular PA | Primary and secondary school students 6 to18 years old | PA, BMI and PF | The shortest was six weeks and the longest was five years. | 18 out of 19 studies reported significant improvements in at least one outcome related to obesity, PA, or PF. The review highlights the potential of school-based interventions to positively impact obesity prevention and the promotion of PA and fitness, particularly through programmes focused on PA. |
| Larouche et al., 2018  [29] | Effectiveness of active school transport interventions: a systematic review and update | RCTs, quasi-experimental, observational studies, including pre-post designs without control groups and retrospective case-control studies. | 30 | Active school transport intervention (AST) | school-aged children and adolescents (6–18-year-olds). | Change in PA | One year to three years | 13 studies resulted in a statistically significant increase in AST, while 8 found no changes, and 5 did not report inferential statistics. The review underscores a considerable variation in intervention outcomes and suggests that multi-component interventions (combining education and encouragement,) tend to be more effective, though the overall quality of evidence was rated as low. |
| Hanna et al., 2023  [20] | A Systematic Review of the Implementation and Effectiveness of ‘The Daily Mile’ on Markers of Children’s Health | Experimental and observational studies.  Mixed-method approaches combining experimental and observational designs | 16 | The Daily Mile Intervention (TDM) | Primary school children  6 to11 years old | Cardiorespiratory fitness, anthropometry and body composition, PA, cognition, and process evaluation | The shortest was three months and the longest was 12 months. | TDM positively impacts cardiorespiratory fitness and PA among primary school children. Despite its benefits, implementation barriers such as TDM's repetitive nature, time constraints, and inadequate facilities were noted, requiring schools to adapt the original TDM format for effective application. |
| Masini et al., 2020  [21] | Evaluation of school-based interventions of active breaks in primary schools: A systematic review and meta-analysis | RCTs, c-RCTs.  Observational studies, including cohort studies. | 28 | Active Break Intervention (AB)  Daily time dedicated to AB was 1- to 15 minutes. | Primary school children  6–12 years old | PA, Classroom Behaviour, Cognitive Function, and Academic Achievement | The shortest was nine weeks and the longest was 6 months. | Active Break interventions in primary schools significantly increased PA levels, specifically MVPA and step count, while also improving classroom behaviour, particularly time on task. However, the effects on cognitive functions and academic achievements were inconclusive, suggesting that the impact of AB may be more pronounced with curriculum-focused activities and active lessons that integrate key learning aspects. |
| Moeini et al., 2021  [30] | Effect of interventions based on regular physical activity on weight management in adolescents: a systematic review and a meta-analysis | Eight were RCTs, one non-RCT, four CCTs and one experimental study | 14 | Educational interventions | Children and adolescents aged 6 - 18 years old. | Change in BMI and the secondary consequence is the promotion of PA | The shortest was 8 weeks and the longest was 6 months. | Interventions led to a statistically significant weight loss of 1.02 kg compared to the control group (95% confidence interval: -4.794 to 0.222). The review underscores the effectiveness of these interventions in improving PA levels among adolescents, thereby contributing to weight management and loss. |
| Wong et al., 2021  [22] | Interventions to Increase Moderate-to-Vigorous Physical Activity in Elementary School Physical Education Lessons: Systematic Review | Three quasi-experimental studies and two RCTs | 5 | Born to Move PA and fitness intervention alongside a regular PE lesson.  Intervention based on self-determination theory and the socio-ecological model | Children aged 8 to 11 years | Fitness infusions and physically active games | The shortest was six weeks and the longest was four years | Finding a pooled mean difference of +14.3% of lesson time spent in MVPA between intervention and control groups. This significant increase suggests that targeted interventions in PE lessons can effectively enhance children's PA levels during school hours. |
| Vaquero-Solís et al., 2020  [31] | School-Based Physical Activity Interventions in Children and Adolescents: A Systematic Review | Quasi-experimental,  c-RCTs, Longitudinal studies | 45 | Interventions based on motivation for the promotion of physical activity and psychosocial benefits | Children and adolescents aged 6 - 18 years old. | Any physical or psychosocial outcome | The shortest was two weeks and the longest was 9 months | 23 studies showed psychological effects post-intervention and 10 studies demonstrated psychosocial impacts. The review emphasizes the importance of motivational processes for engaging in PA and sports as precursors to psychosocial changes, highlighting the role of strategies and the duration of studies in maintaining significant changes over time. |
| Breslin et al., 2023  [23] | A systematic review of the effect of The Daily Mile™ on children’s physical activity, physical health, mental health, well-being, academic performance and cognitive function | RCTs, quasi-experimental studies, pilot studies, repeated measures studies, cross-sectional studies, and natural experiments. | 13 | The Daily Mile Intervention (TDM) | school-aged children (aged 4–12 years) | PA levels (self-report or objective measures). Physical fitness. Indicators of physical health status. mental health, psychological well-being, academic performance, and cognitive function | The shortest was one session of TDM and the longest was 12 months | A positive impact on increasing MVPA and PF in children, though it did not significantly affect BMI or academic performance. Notably, longer-term participation in TDM was associated with improvements in visual-spatial working memory and short-term mental health benefits, but no significant effects were observed on overall well-being or cognitive function. |
| Schönbach et al., 2020  [32] | School-based interventions to promote active school transportation by bicycle among Children and adolescents: A Systematic review | RCTs and non-RCTs | 9 | An intervention aimed at promoting active school travel by bicycle | Primary and secondary school students of all ages | PA levels, PF, weight status, active travel and cycling skills | The shortest was four weeks and the longest was one year. | A lack of consistent significant improvements across the included studies. While some interventions, like the voluntary bicycle train accompanied by adults, showed promise in increasing cycling trips to school and improving psychosocial factors like parental and child self-efficacy, the overall evidence was limited by the weak methodological quality of the included studies. |
| Buru et al., 2020  [18] | The Efficacy of School-Based Interventions in Preventing Adolescent Obesity in Australia | RCTs and quasi-experimental designs | 13 | Use of height-adjustable desks to reduce classroom sitting time.  PA interventions | Adolescents (12 to 18 years old) | WC, Body fat, Body weight, Lean Mass, Energy expenditure, PA, screen time, Nutritional behaviour | The shortest was four months and the longest was two years with a follow-up of three years. | This review showed varying effectiveness. Multi-component interventions aligned with theoretical frameworks like Social Cognitive Theory and incorporating physical activity, dietary changes, and reductions in sedentary behaviour demonstrated promising results in managing obesity among adolescents. |
| Brandes et al., 2022  [24] | A scoping review on characteristics of school-based interventions to promote physical activity and cardiorespiratory fitness among 6- to 10-year-old children | c-RCTs and non-RCTs | 192 | Most of the interventions were theory-based | School children aged 6 to 10 years | PA, cardiorespiratory fitness, sedentary behaviors. | Intervention duration on average was 44 weeks (Range: 1–410 weeks) | It found that interventions frequently focused on single aspects, with "Health skills and education" being the most common feature, and highlighted that theory-based interventions, either single or multi-feature, showed potential for improving PA, cardiorespiratory fitness, and sedentary behaviour outcomes. |
| Owen et al., 2017  [19] | The effectiveness of school-based physical activity interventions for adolescent girls: A systematic review and meta-analysis | Fourteen RCTs, including three c-RCTs and one pilot RCT.  Five quasi-experimental studies.  One case-crossover study | 20 | Most of them were multi-component interventions.  Four of them were modified PE lessons.  Two were educational-based interventions | Adolescent girls aged 11 to 18 years. | Objective measure or self-reported PA levels. | The shortest was six months and the longest was three years. Only two studies have a long-term follow-up (i.e., ≥12 months) | A small but significant effect of school-based PA interventions on increasing activity levels among adolescent girls (g = 0.37, p < 0.05), which diminished to a very small effect after outlier removal (g = 0.07, p = 0.05). The study highlights the challenges of modifying PA behaviours in adolescent girls through school programs. It suggests multi-component interventions and those grounded in theory may offer slightly more effective approaches, although overall impacts are modest. |
| Chavez & Nam, 2020  [17] | School-based obesity prevention interventions in Latin America: A systematic review | RCTs, non-RCTs, and quasi-experimental studies. | 16 | Multi-component school-based interventions | School children aged 6 to 18 years | BMI, PA levels, Z-score, | The shortest was four months and the longest was two years, with the shortest follow-up was five months and the longest one was two years. | Multi-component interventions which incorporated health education and parental involvement with a focus on healthy eating and PA, were most effective. |
| Masini et al., 2022  [27] | Evaluation of feasibility, effectiveness, and sustainability of school-based physical activity “active break” interventions in pre-adolescent and adolescent students: a systematic review | Two studies were RCTs and one was quasi-experimental study | 3 | Physically active lessons and active breaks. | Secondary and high school-aged 12 to 18 years | Objective measure for PA level or PF; or cognitive functions or classroom behaviours or quality of life or well-being. | The shortest was 11 weeks and the longest was 7 months. | It found that such interventions are feasible and acceptable, with two studies showing positive effects on students' classroom behaviour and quality of life, and one study noting an increase in school PA levels. However, this increase was not observed in overall PA levels or in the reduction of sedentary behaviour. |
| Carrasco-Uribarren et al., 2023  [25] | Improvement of In-School Physical Activity with Active School-Based Interventions to Interrupt Prolonged Sitting: A Systematic Review and Meta-Analysis | Clinical trials. | 9 | Active-break intervention | Children between 6 and 12 years old | PA, MVPA, SB | The shortest was 5 days and the longest was 3 years. | Active-break interventions in schools significantly improved PA and MVPA levels among children aged 6 to 12 years during school time. However, these interventions did not significantly reduce sedentary behaviour, highlighting the need for additional strategies to address prolonged sitting in school settings. |
| Nally et al., 2021  [26] | The Effectiveness of School-Based Interventions on Obesity-Related Behaviours in Primary School Children: A Systematic Review and Meta-Analysis of Randomised Controlled Trials | RCTs,  c-RCTs. | 48 | Most of them were multi-component interventions. | Children between 6 and 11 years old. | Changes in obesity-related behaviours, including increased PA, decreased sedentary behaviour, and improved nutrition behaviour, as well as changes in BMI or BMI z-score | The shortest was 12 weeks and the longest was 4 years. | It found small positive effects on PA and significant reductions in both BMI and BMI z-score, showcasing their potential effectiveness in obesity prevention. However, there was no significant effect observed on sedentary behaviour, energy intake, or fruit and vegetable intake, highlighting areas for future improvement.  Top of Form |
| Guirado et al., 2021  [33] | Effects of Classroom Active Desks on Children and Adolescents’ Physical Activity, Sedentary Behavior, Academic Achievements and Overall, Health: A Systematic Review | RCTs, non-RCT, cross-over design studies | 23 | Corresponds to standing desk, sit-to-stand desk, or stand-biased desk. (Upright active desk), (Cycling desk), (Stability ball). | Children and adolescents aged 5–17 years | Body composition, SB and PA, energy expenditure, cognitive and academic performance and PF. | The shortest was two weeks and the longest was two years. | An increase of around 36% in energy expenditure for cycling desks and between 15% and 27.7% for upright active desks. Children increased inhibitory control and selective attention capacity while using cycling desks. active desks appear to be a promising intervention in classrooms to improve health-related outcomes |
| Campos‐Garzón et al., 2023  [34] | Contribution of active commuting to and from school to device-measured physical activity levels in young people: A systematic review and meta-analysis | 11 studies had a cross-sectional design, two were longitudinal and one was  c-RCT. | 14 | Active commuting to and from school (ACS) | Children and adolescents aged 6 to 18 years | Accelerometer to measure PA. | Not reported | They found that ACS could contribute about 48% of the daily PA recommendations if both directions (home-school and school-home) are actively performed, emphasising the significant role of ACS in enhancing PA levels among the youth. Moreover, the study highlighted the need for future research to focus on promoting ACS as a means to increase daily PA levels in the young population. |
| Alalawi et al., 2023  [16] | School-based physical activity interventions among children and adolescents in the Middle East and Arabic speaking countries: A systematic review | Thirteen RCTs and four non-RCTs (quasi-experimental designs) | 17 | Most of the interventions were multi-component (lifestyle, diet, education).  Few were on PA. | Children and adolescents aged 6 to 18 years | Self-reported or objectively measured change in PA levels. | The shortest was two months and the longest was 3 years. | Eleven studies reported statistically significant improvements in the levels of PA among their participants. Based largely on self-reported outcomes, increases in PA between 58% and 72% were reported. The studies with a follow-up period greater than three months reported sustained PA levels. |
| Bermejo-Cantarero et al., 2023  [35] | Are Physical Activity Interventions Effective in Improving Health-Related Quality of Life in Children and Adolescents? A Systematic Review and Meta-Analysis | 15 studies were RCTs and two were quasi-experimental designs | 17 | Most of them were adding extra (PE) lessons to the regular school curriculum.  Few of them were short active breaks | Children and adolescents aged 6 to 18 years. | The effects of PA interventions on various domains of HRQoL in healthy children and adolescents. These domains included total HRQoL score, physical well-being, psychological well-being, autonomy and parent relation, social support and peers, and the school environment. | The length of interventions ranged from 2 to 12 months.  Four studies conducted long-term follow-up ranging from 3 months to 3 years. | PA interventions were found to be an effective strategy for improving overall health-related quality of life (HRQoL) and its significant domains, such as physical and psychological well-being, autonomy and parent relation, and social support and peers, in children and adolescents. However, the interventions did not significantly impact the school environment domain of HRQoL. |
| Pfledderer et al., 2021  [36] | School-based physical activity interventions in rural and urban/ suburban communities: A systematic review and meta-analysis | RCTs, non-RCTs and CCTs. | 33 | The intervention component was physical education utilized 20 times.  Classroom-based interventions were utilized 16 times. | Children and adolescents aged 6 to 18 years. | PA | The length of interventions ranged from six weeks to two years. | A significant increase in total PA among participants, with school-based interventions demonstrating a positive effect across both rural and urban/suburban settings, suggests the effectiveness of such programmes in promoting PA among youth regardless of geographic location. |
| Ahmed et al., 2021  [15] | The effectiveness of physical activity interventions in Asian children and adolescents: a systematic review | RCTs,  c-RCTs,  non-RCTs. | 30 | Most of them used a theoretical framework/ model. | Children and adolescents aged 3 to 18 years. | Change in PA behaviours | The duration of intervention ranged from one day to 60 months (median 4.5 months) | 21 studies of moderate-to-high quality demonstrated significant increases in PA behaviour or PF. The review suggests that school-based, short-term interventions with either single or multi-component approaches, including PA sessions, PE, health education, and/or educational materials, may effectively increase overall PA in Asian children and adolescents. |
| Neil-Sztramko et al., 2021  [37] | School-based physical activity programs for promoting physical activity and fitness in children and adolescents aged 6 to 18 (Review) | RCTs including both individual-level RCTs and c-RCTs, | 77 | 40 multi-component interventions.  19 school time physical activity.  15 enhanced physical education | Children and adolescents 6 to 18 years old | Objective measure of PA or fitness. (Proportion of participants meeting PA guidelines and duration of MVPA and sedentary time). | The duration of interventions varied greatly from a minimum of 12 weeks to 6 years, with 10 studies reporting intervention periods of 3 years or longer. | Such interventions likely result in a modest increase in MVPA by approximately 0.73 minutes per day, with low to moderate certainty of evidence. Additionally, the review suggests these programmes may slightly decrease sedentary time by about 3.78 minutes per day and improve PF, as indicated by maximal oxygen uptake, yet with a very small and uncertain impact on BMI. |

**S4 Appendix: Quality assessment by AMSTAR version 2**

| # | **Author, year** | **1** | **2** | **3** | **4** | **5** | **6** | **7** | **8** | **9** | **10** | **11** | **12** | **13** | **14** | **15** | **16** | **Evaluation final rate** |
| --- | --- | --- | --- | --- | --- | --- | --- | --- | --- | --- | --- | --- | --- | --- | --- | --- | --- | --- |
| 1 | Yuksel et al., 2020 | X | X | X | Y | Y | Y | X | Y | Y | X | NA | NA | / | / | / | X | Critically low |
| 2 | Larouche et al., 2018 | Y | / | X | Y | Y | Y | X | Y | Y | X | NA | NA | Y | / | NA | / | Critically low |
| 3 | Hanna et al., 2023 | X | / | Y | / | X | X | X | Y | X | X | NA | NA | Y | Y | / | / | Critically low |
| 4 | Masini et al., 2020 | Y | Y | Y | Y | Y | Y | X | Y | Y | X | Y | / | / | Y | Y | / | High |
| 5 | Moeini et al., 2021 | Y | Y | Y | Y | Y | Y | X | Y | Y | X | Y | Y | Y | Y | Y | Y | High |
| 6 | Wong et al., 2021 | Y | Y | Y | Y | X | X | X | Y | Y | X | / | / | Y | / | Y | X | Low |
| 7 | Vaquero-Solís et al., 2020 | Y | X | X | / | / | / | X | X | Y | X | NA | NA | Y | / | NA | / | Critically low |
| 8 | Breslin et al., 2023 | Y | Y | Y | Y | Y | X | Y | / | Y | X | NA | NA | / | / | NA | / | Low |
| 9 | Schönbach et al., 2020 | Y | X | Y | Y | Y | Y | / | Y | Y | X | NA | NA | / | / | NA | / | Low |
| 10 | Buru et al., 2020 | X | X | X | Y | Y | X | / | Y | Y | X | NA | NA | X | / | / | X | Critically low |
| 11 | Brandes et al., 2022 | Y | / | Y | Y | / | / | Y | Y | X | X | NA | NA | X | X | X | / | Critically low |
| 12 | Owen et al., 2017 | X | / | X | X | X | / | X | Y | / | X | / | / | / | / | / | / | Low |
| 13 | Chavez & Nam, 2020 | X | X | X | X | X | X | / | / | Y | X | NA | NA | / | Y | NA | X | Critically low |
| 14 | Masini et al., 2022 | Y | Y | Y | Y | Y | Y | / | Y | Y | X | NA | NA | / | / | / | / | High |
| 15 | Carrasco-Uribarren et al., 2023 | X | Y | Y | Y | Y | / | / | Y | Y | X | Y | / | / | / | Y | / | High |
| 16 | Nally et al., 2021 | X | Y | Y | Y | Y | Y | / | Y | Y | X | Y | Y | / | / | / | Y | High |
| 17 | Guirado et al., 2021 | X | Y | Y | Y | Y | Y | / | Y | Y | X | NA | NA | Y | / | / | X | Moderate |
| 18 | Campos‐Garzón et al., 2023 | Y | Y | Y | Y | Y | / | / | Y | / | X | / | / | / | / | X | / | Moderate |
| 19 | Alalawi et al., 2023 | Y | / | X | Y | Y | X | Y | Y | Y | X | NA | NA | / | Y | X | X | Moderate |
| 20 | Bermejo-Cantarero et al., 2023 | X | / | Y | Y | Y | Y | / | Y | Y | X | Y | Y | / | / | X | X | Moderate |
| 21 | Pfledderer et al., 2021 | Y | / | Y | / | Y | X | / | Y | X | X | / | / | X | Y | Y | X | Low |
| 22 | Ahmed et al., 2021 | Y | / | Y | Y | Y | Y | Y | Y | Y | X | NA | NA | / | / | / | X | High |
| 23 | Neil-Sztramko et al., 2021 | Y | Y | / | / | / | / | / | / | / | Y | NA | NA | Y | Y | X | / | High |

***Value of each item****

| *“Y” Yes* | *“X” No* | ***“/”*** *Partial yes* | ***“NA”*** *Not applicable* |
| --- | --- | --- | --- |

***Items of AMSTAR 2*****

1. *Did* *the research questions and inclusion criteria for the review* *include the components of PICO?*
2. *Did the report of the review contain an explicit statement that the review methods were established prior to the conduct of the review and did the report justify any significant deviations from the protocol?*
3. *Did the review authors* *explain their selection of the study designs for inclusion in the review?*
4. *Did the review authors use a comprehensive literature search strategy?*
5. *Did the review* *authors perform study selection in duplicate?*
6. *Did the review authors perform* *data extraction in duplicate?*
7. *Did the review authors* *provide a list of excluded studies and justify the exclusions?*
8. *Did the review authors describe the included studies in adequate detail?*
9. *Did the review authors use a satisfactory technique for* *assessing the risk of bias (RoB) in individual studies that were included in the review?*
10. *Did the review authors* *report on the sources of funding for the studies included in the review?*
11. *If meta-analysis was performed did the review authors use* *appropriate methods for statistical combination of results?*
12. *If meta-analysis was performed, did the review authors* *assess the potential impact of RoB in individual studies on the results of the meta-analysis or other evidence synthesis?*
13. *Did the review authors* *account for the risk of bias in individual studies when interpreting/ discussing the results of the review?*
14. *Did the review authors provide a satisfactory explanation for, and discussion of, any heterogeneity observed in the results of the review?*
15. *If they performed quantitative synthesis did the review authors carry out an adequate investigation of publication bias (small study bias) and discuss its likely impact on the results of the review?*
16. *Did the review authors report any potential sources of conflict of interest, including any funding they received for conducting the review?*

***Evaluation of the final rate***

| ***High*** | *No or one non-critical weakness: the systematic review provides an accurate and comprehensive summary of the results of the available studies that address the question of interest.* |
| --- | --- |
| ***Moderate*** | *More than one non-critical weakness*: the systematic review has more than one weakness but no critical flaws. It may provide an accurate summary of the results of the available studies that were included in the review.* |
| ***Low*** | *One critical flaw with or without non-critical weaknesses: the review has a critical flaw and may not provide an accurate and comprehensive summary of the available studies that address the question of interest.* |
| ***Critically low*** | *More than one critical flaw with or without non-critical weaknesses: the review has more than one critical flaw and should not be relied on to provide an accurate and comprehensive summary of the available studies.* |
