# Supplementary material for: Umbrella review of international evidence for the effectiveness of school-based physical activity interventions
Source: PLoS One. 2024 Jun 13;19(6):e0304513. doi: 10.1371/journal.pone.0304513 (PMC11175402; doi:10.1371/journal.pone.0304513)
Supplement: Attachment — Submitted filename: PONE-D-24-10547_reviewer.pdf [file pone.0304513.s003.pdf]

PLOS ONE

The Effectiveness of School-Based Interventions to Increase Physical Activity among Children and Young People aged 6 to 18 years: A Systematic Review of Reviews

--Manuscript Draft--

**Manuscript Number:**	PONE-D-24-10547
**Article Type:**	Research Article
**Full Title:**	The Effectiveness of School-Based Interventions to Increase Physical Activity among Children and Young People aged 6 to 18 years: A Systematic Review of Reviews
**Short Title:**	The Effectiveness of School-Based Interventions to Increase Physical Activity Globally
**Corresponding Author:**	Abdullah Alalawi, Ph.D.<br>The University of Sheffield<br>Sheffield, UNITED KINGDOM
**Keywords:**	Keywords: systematic review; health promotion; school-based; physical activity interventions
**Abstract:**	Abstract:<br>Background: Obesity and physical inactivity among children and young people are public health concerns. Despite the wide variety of interventions available to promote physical activity, little is known about which interventions are most effective. This review aimed to evaluate the existing literature on school-based interventions that aim to increase physical activity among children and young people aged 6 to 18 years.<br>Methods: A systematic review of reviews was undertaken. We searched for systematic reviews and meta-analyses published between December 2017 and January 2024 using databases such as PubMed, Scopus, and CINAHL. Titles and abstracts were independently screened by two reviewers, who also conducted data extraction and quality assessments. We focused on outcomes like changes in physical activity levels and body mass index to assess the effectiveness of the interventions.<br>Results: A total of 23 reviews examining school-based physical activity interventions met the inclusion criteria, comprising 15 systematic reviews and 8 meta-analyses. All reviews (N=23) were implemented in the school setting: three in primary schools, seven in secondary schools, and thirteen targeted both primary and secondary schools. The findings demonstrated that six reviews reported a statistical increase in physical activity levels among the target population, and one review found a decrease in body mass index. The most promising interventions focused on physical activity included within the school curriculum and were characterised as long-term interventions.<br>Conclusion: Some interventions were promising in promoting physical activity among school-aged children and young people such as Daily Mile, Active Break, and Active transport while multi-component interventions seem to be positively effective in reducing BMI. Future efforts should focus on long-term, theory-driven programmes to ensure sustainable increases in physical activity.
**Order of Authors:**	Abdullah Omar Alalawi, Ph.D.
	Lindsay Blank
	Elizabeth Goyder, Professor
**Additional Information:**	
**Question**	**Response**
**Financial Disclosure**<br><br>Enter a financial disclosure statement that describes the sources of funding for the work included in this submission. Review the submission guidelines for detailed requirements. View published research	The author(s) received no specific funding for this work.

Powered by Editorial Manager® and ProduXion Manager® from Aries Systems Corporation

articles from *PLOS ONE* for specific examples.<br><br>This statement is required for submission and **will appear in the published article** if the submission is accepted. Please make sure it is accurate.<br><br>**Funded studies**<br>Enter a statement with the following details:<br>• Initials of the authors who received each award<br>• Grant numbers awarded to each author<br>• The full name of each funder<br>• URL of each funder website<br>• Did the sponsors or funders play any role in the study design, data collection and analysis, decision to publish, or preparation of the manuscript?<br><br>Did you receive funding for this work?	
**Competing Interests**<br><br>Use the instructions below to enter a competing interest statement for this submission. On behalf of all authors, disclose any competing interests that could be perceived to bias this work—acknowledging all financial support and any other relevant financial or non-financial competing interests.<br><br>This statement is required for submission and **will appear in the published article** if the submission is accepted. Please make sure it is accurate and that any funding sources listed in your Funding Information later in the submission form are also declared in your Financial Disclosure statement.<br><br>View published research articles from *PLOS ONE* for specific examples.	The authors have declared that no competing interests exist.

**NO authors have competing interests**<br><br>Enter: *The authors have declared that no competing interests exist.*<br><br>**Authors with competing interests**<br><br>Enter competing interest details beginning with this statement:<br><br>*I have read the journal's policy and the authors of this manuscript have the following competing interests: [insert competing interests here]*<br><br>* typeset	
**Ethics Statement**<br><br>Enter an ethics statement for this submission. This statement is required if the study involved:<br><br>• Human participants<br>• Human specimens or tissue<br>• Vertebrate animals or cephalopods<br>• Vertebrate embryos or tissues<br>• Field research<br><br>Write "N/A" if the submission does not require an ethics statement.<br><br>General guidance is provided below. Consult the submission guidelines for detailed instructions. **Make sure that all information entered here is included in the Methods section of the manuscript.**	"N/A"

Powered by Editorial Manager® and ProduXion Manager® from Aries Systems Corporation

**Format for specific study types**<br><br>**Human Subject Research (involving human participants and/or tissue)**<br>• Give the name of the institutional review board or ethics committee that approved the study<br>• Include the approval number and/or a statement indicating approval of this research<br>• Indicate the form of consent obtained (written/oral) or the reason that consent was not obtained (e.g. the data were analyzed anonymously)<br><br>**Animal Research (involving vertebrate animals, embryos or tissues)**<br>• Provide the name of the Institutional Animal Care and Use Committee (IACUC) or other relevant ethics board that reviewed the study protocol, and indicate whether they approved this research or granted a formal waiver of ethical approval<br>• Include an approval number if one was obtained<br>• If the study involved *non-human primates*, add *additional details* about animal welfare and steps taken to ameliorate suffering<br>• If anesthesia, euthanasia, or any kind of animal sacrifice is part of the study, include briefly which substances and/or methods were applied<br><br>**Field Research**<br><br>Include the following details if this study involves the collection of plant, animal, or other materials from a natural setting:<br>• Field permit number<br>• Name of the institution or relevant body that granted permission	
**Data Availability**<br><br>Authors are required to make all data underlying the findings described fully available, without restriction, and from the time of publication. PLOS allows rare exceptions to address legal and ethical concerns. See the PLOS Data Policy and FAQ for detailed information.	Yes - all data are fully available without restriction

A Data Availability Statement describing where the data can be found is required at submission. Your answers to this question constitute the Data Availability Statement and **will be published in the article**, if accepted.<br><br>**Important:** Stating 'data available on request from the author' is not sufficient. If your data are only available upon request, select 'No' for the first question and explain your exceptional situation in the text box.<br><br>Do the authors confirm that all data underlying the findings described in their manuscript are fully available without restriction?	
**Describe where the data may be found in full sentences. If you are copying our sample text, replace any instances of XXX with the appropriate details.**<br><br>• If the data are **held or will be held in a public repository**, include URLs, accession numbers or DOIs. If this information will only be available after acceptance, indicate this by ticking the box below. For example: *All XXX files are available from the XXX database (accession number(s) XXX, XXX.).*<br>• If the data are all contained **within the manuscript and/or Supporting Information files**, enter the following: *All relevant data are within the manuscript and its Supporting Information files.*<br>• If neither of these applies but you are able to provide **details of access elsewhere**, with or without limitations, please do so. For example:<br><br>*Data cannot be shared publicly because of [XXX]. Data are available from the XXX Institutional Data Access / Ethics Committee (contact via XXX) for researchers who meet the criteria for access to confidential data.*<br><br>*The data underlying the results presented in the study are available from (include the name of the third party*	All relevant data are within the manuscript and its Supporting Information files.

and contact information or URL).<br>• This text is appropriate if the data are owned by a third party and authors do not have permission to share the data.<br><br>* typeset	
Additional data availability information:	

Powered by Editorial Manager® and ProduXion Manager® from Aries Systems Corporation

Manuscript Click here to access/download;Manuscript;Manuscript.docx

The Effectiveness of School-Based Interventions to Increase Physical Activity among Children and Young People aged 6 to 18 years: A Systematic Review of Reviews

Abdullah Alalawi[1,2*], Lindsay Blank[3], Elizabeth Goyder[3]

[1] Health and Related Research, University of Sheffield, Sheffield, United Kingdom.

[2] Al Qunfudah Health Sciences College, Umm Al-Qura University, Makkah, Saudi Arabia.

[3] Health and Related Research, University of Sheffield, Sheffield, United Kingdom.

* Correspondence: aoalalawi1@sheffield.ac.uk

Abstract:

Background: Obesity and physical inactivity among children and young people are public health concerns. Despite the wide variety of interventions available to promote physical activity, little is known about which interventions are most effective. This review aimed to evaluate the existing literature on school-based interventions that aim to increase physical activity among children and young people aged 6 to 18 years.

Methods: A systematic review of reviews was undertaken. We searched for systematic reviews and meta-analyses published between December 2017 and January 2024 using databases such as PubMed, Scopus, and CINA[illegible]. Titles and abstracts were independently screened by two reviewers, who also conducted data extraction and quality assessments. We focused on outcomes like changes in physical activity levels and body mass index to assess the effectiveness of the interventions.

Results: A total of 23 reviews examining school-based physical activity interventions met the inclusion criteria, comprising 15 systematic reviews and 8 meta-analyses. All reviews (N=23) were implemented in the school setting: three in primary schools, seven in secondary schools, and thirteen targeted both primary and secondary schools. The findings demonstrated that six reviews reported a statistical increase in physical activity levels among the target population, and one review found a decrease in body mass index. The most promising interventions focused on physical activity included within the school curriculum and were characterised as long-term interventions.

Conclusion: Some interventions were promising in promoting physical activity among school-aged children and young people such as Daily Mile, Active Break, and Active transport while multi-component interventions seem to be positively effective in reducing BMI. Future efforts

should focus on long-term, theory-driven programmes to ensure sustainable increases in physical activity.

Keywords: systematic review; health promotion; school-based; physical activity interventions

Introduction

The World Health Organization (WHO) identifies childhood obesity as a significant public health concern in the 21st century. In 2016, a staggering 41 million children below the age of five were reported to be overweight, predominantly residing in developing nations [1]. Overweight and obesity are linked to various health repercussions that frequently manifest later in life. Consequently, children who are overweight or obese are at an increased risk of developing non-communicable diseases like diabetes, cardiovascular diseases, and metabolic syndrome in adulthood [2,3]. One of the essential factors for preventing obesity encompasses maintaining proper engagement in regular physical activity. The World Health Organization advises that individuals aged 5–17 years should engage in a minimum of sixty minutes of moderate to vigorous intensity physical activity (MVPA) each day, with a primary focus on aerobic activities [4].

Childhood overweight and obesity have been associated with adverse psychosocial outcomes, including issues such as poor self-image, low self-esteem, an elevated risk of developing eating disorders, and a diminished quality of life [5]. The rising occurrence of childhood obesity for example in Europe can be attributed to societal and lifestyle shifts observed over the past three decades [6]. These changes are marked by the adoption of unhealthy eating habits, characterized by a high consumption of unhealthy fats and added sugars, coupled with a reduced intake of complex carbohydrates and fiber. Additionally, a sedentary lifestyle, characterized by prolonged screen time and a lack of involvement in physical activities, has contributed to this trend [7]. To the best of our knowledge, numerous programs have been developed to promote healthy lifestyles and prevent obesity in children. The predominant approach in the vast majority of these initiatives involves utilizing schools as the primary setting for implementing interventions targeted at school-aged children [8,9].

Schools play a significant role in shaping the social environment for children, and various efforts have been undertaken to leverage this setting for promoting healthy behaviors among youth, encompassing physical activity habits [10,11]. Programs implemented in schools can reach nearly all children, spanning various ethnic and socio-economic backgrounds. Moreover, schools can encourage physical activity through structured programs, with sports education being a compulsory component of the school curriculum. In many instances, children are also required to walk to and from school on weekdays [12]. Consequently, schools serve as an ideal environment to implement PA interventions [10].

Previous umbrella systematic review [9] synthesized evidence on interventions to increase physical activity among children and young people across various settings, with a significant focus on school-based programmes. It finds that interventions involving physical activity embedded within the school curriculum, long-term initiatives, teacher involvement, and family support show the most promise in enhancing physical activity levels. The review includes studies conducted in school, preschool, family, and community settings, highlighting the comprehensive scope of its analysis and the pivotal role of schools in promoting physical activity.

This umbrella systematic review aims to update and provide a comprehensive overview of the latest studies on the effectiveness of school-based physical activity interventions to promote physical activity among children, and young people aged 6 to 18 years old worldwide.

Methods

An umbrella systematic review incorporating literature search and narrative synthesis was conducted to identify, evaluate, and synthesize relevant evidence from intervention studies conducted in school settings globally. This review was conducted in accordance with the PRISMA guidelines for systematic reviews. The review protocol was registered with the

PROSPERO International Prospective Register of Systematic Reviews, under registration number CRD42023494953.

Formulation of Research Question

The PICO framework was considered to formulate the aim of this research by describing the population (P), intervention (I), comparison (C) and outcome (O), as summarised in Table 1 below. The value of the PICO framework is that it breaks down complex questions into simpler phrases that also form the inclusion and exclusion criteria (Fenton & Baxter, 2016).

Table 1: PICO Framework

**Population**	School children and young people aged 6–18 years.
**Intervention**	School-based PA as either a primary intervention or a component of a multi behavioural intervention
**Comparison**	No intervention or usual school provision of PA, comparison of two or more interventions.
**Outcome**	The primary outcome is the self-reported or objectively measured change in PA levels. Secondary outcome includes changes in Body Mass Index.

Inclusion and Exclusion Criteria

Ideally, including a large body of evidence is desirable because it provides more material from which to derive decisions. However, given the extensive volume and diversity of research on school-based physical activity interventions worldwide, we decided to conduct an umbrella review of relevant systematic reviews, which included primary studies on the effectiveness of these interventions. Consequently, we applied the following inclusion and exclusion criteria to select the most appropriate articles for inclusion in this umbrella review.

Table 2: Inclusion and Exclusion Criteria

Inclusion criteria	Exclusion criteria
Systematic reviews and meta-analyses that incorporated various study designs, including randomized controlled trials (RCTs), cluster-randomized controlled trials (c-RCT), controlled trials (CT), quasi-experimental studies, and observational studies	Studies that did not adopt a systematic review approach, narrative reviews, and primary studies.
Reviews involved participants between the ages (6 to 18 years).	Reviews discussing physical activity without incorporating an intervention
Studies that explored at least one intervention for increasing physical activity among the targeted population	Reviews involving interventions unrelated to physical activity and movement.
Interventions that were implemented in schools and quantitative evaluations of PA	Reviews included interventions not based on schools
Reviews based on a search conducted from 2018 onwards because there was previously an umbrella review based on a search for studies published from January 2010 until November 2017 [9]	
Studies published in Arabic or English language.	

Literature Search Process

Systematic searches were conducted in PubMed/MEDLINE, Scopus and CINAHL. These databases were chosen not only because they are easily accessible but also because they contain

a large volume of systematic reviews and meta-analysis, due to their public health scope. Furthermore, we searched reference lists in included reviews to identify relevant studies. Details of the search strategy are outlined in the (S1 Appendix of the S1 File).

Studies Selection

All records were exported to Mendeley reference manager software. Duplicate records and all studies whose titles or abstracts did not meet the inclusion criteria were excluded. Screening of studies was conducted by two independent reviewers and, titles and abstracts of records retrieved from searches were screened for inclusion, and any disagreement was resolved by discussion with a third reviewer.

Data Extraction

A standardised approach was used to extract data from the selected studies [13], including study details such as the author(s), publication year, title, design, the number of studies included in the reviews, descriptions of interventions, duration of interventions and follow-ups, and outcomes measured, including physical activity levels and BMI, along with the main findings.

Quality Assessment

To evaluate the methodological quality and assess the risk of bias within the included reviews, the updated AMSTAR 2 version was utilised for analysing systematic reviews and meta-analyses, adhering to the recommendations specified by [14]. This tool enabled the systematic reviews to be classified based on their overall quality into four categories: high, moderate, low, or critically low. any disagreement was resolved by discussion with a third reviewer.

Results

This results section contains three main parts: the search outcome, data extraction and synthesis, and quality of included reviews.

Search Outcome

The search was conducted in January 2024. The initial literature search produced a total of 2365 studies: PubMed/MEDLINE 1359, Scopus 290 and CINAHL 716. There were 682 duplicates, and after their exclusion, the 1683 remaining records' titles and abstracts were screened against the inclusion and exclusion criteria. The full texts of the remaining 60 studies were screened which led to the exclusion of 35 studies. The main reasons for exclusion were (1) having an inappropriate target population (n=8); (2) being protocols (n=2); (3) not school-based (n=5); (4) measured different outcomes (n=17) and (5) did not adopt a systematic review approach (n=3). Further articles (n=4) were identified from reference lists of included reviews. Details on excluded studies can be found in the S2 Appendix in the S1 File. A PRISMA flow diagram shows search results (Fig 1).

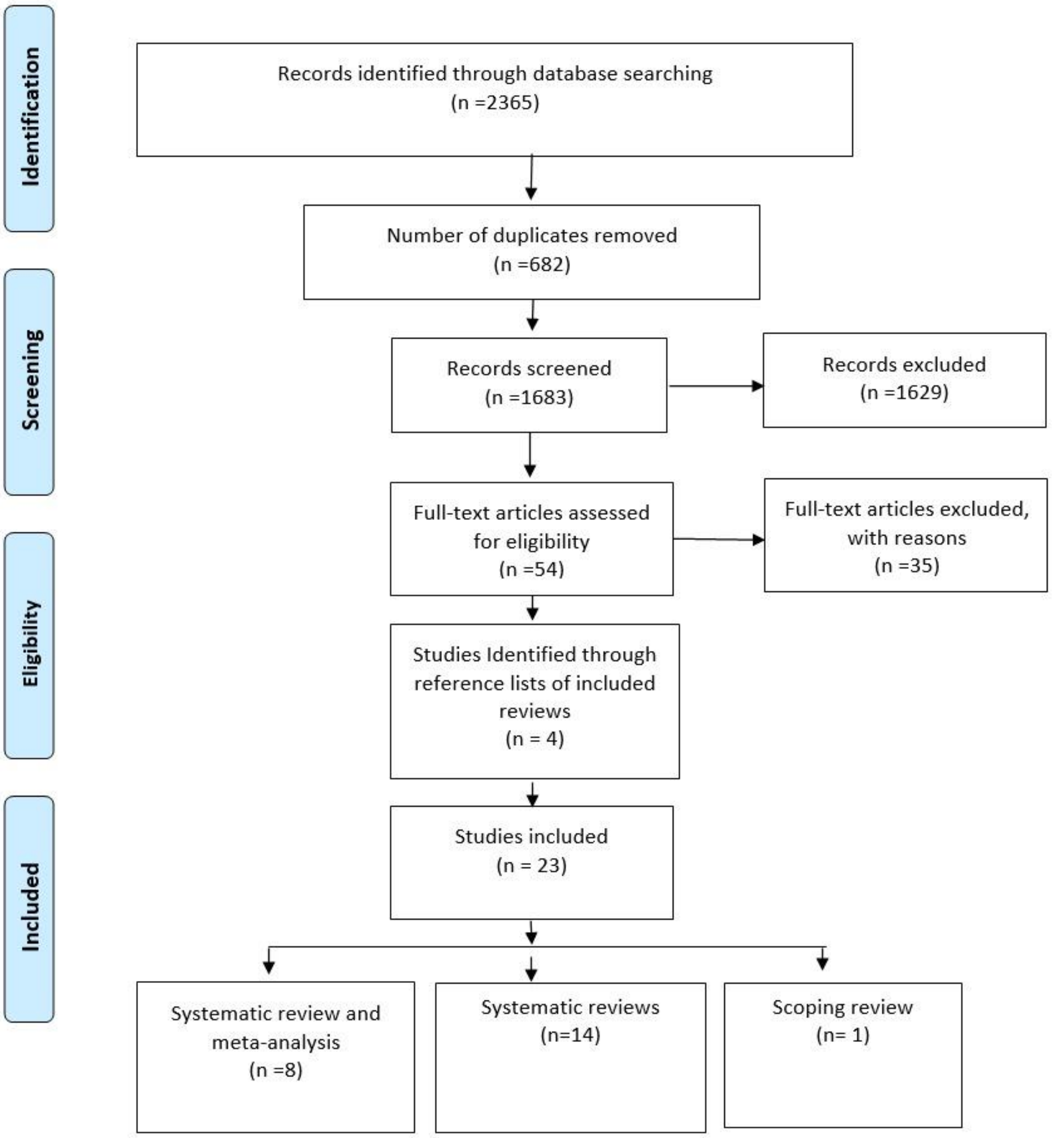

Figure 1: PRISMA Flow Diagram

Review characteristics

A total of 23 studies met the inclusion criteria, comprising 14 systematic reviews, eight meta-analyses, and one scoping review that employed a systematic review methodology. The characteristics of these reviews are detailed in Supplemental Table 2, accessible in S3 Appendix in the S1 File. The majority of the reviews were international in scope, with only four reviews targeting specific regions: one focused exclusively on Asian countries [15], another on the Middle East and Arabic-speaking countries [16], a third on school children in Latin America [17], and a fourth on adolescents in Australia [18]. All reviews included both males and females except one review which targeted only girls [19].

School-Based Interventions

Twenty-three studies were included in this umbrella review, and these studies were grouped according to age and intervention type.

Interventions for Children aged 6 to 12 Years

Seven reviews [20–26] concerned interventions conducted in the primary schools setting for children aged between 6 and 12 years.

Daily Mile Programme: Two reviews [20,23] examined the effectiveness of this intervention, Breslin and their colleagues observed significant variability in outcomes across studies. Specifically, Breslin et al. reported one intervention session led to a significant increase in MVPA, with the intervention group achieving 10.67±2.74 minutes of MVPA compared to 0.44±0.95 minutes in the control group. Another study within the same review [23] noted a significant increase in PA levels after eight months, showing a daily increase of 9.1 minutes of MVPA in the intervention group. Regarding physical fitness, six primary studies in Breslin's review documented a notable improvement in the intervention group versus the control group, although comparing results was challenging due to the varied fitness tests used.

Overall, Breslin and colleagues concluded that sustained participation in the Daily Mile leads to increased MVPA and physical fitness among school-aged children, without significant impacts on academic performance or BMI. Only one study highlighted a short-term enhancement in mental health [23]. Similar results were reported by [20]. However, they also reported some implementation barriers for "The Daily Mile programme" such as repetitive nature, time constraints associated with competing curriculum demands, and inadequate facilities regularly necessitate the adaptation and development of the original Daily Mile intervention format by schools and teachers [20]. It should be noted that Breslin et al review and Hanna et al review included largely the same primary studies and the quality assessment for included studies conducted by Breslin et al revealed that one study was excellent, six were good, five were fair and none of them was poor quality.

Active-break intervention: Two systematic reviews [21,25], evaluated the effectiveness of this intervention, Carrasco-Uribarren et al, found a significant improvement in both PA and MVPA, levels. Six included studies in [25] review, recorded a significant increase in in-school PA (SMD = 0.46; 95% CI: 0.28, 0.64; I2: 52%). Furthermore, five studies recorded a significant increase in MVPA (MD = 3.20; 95% CI: 3.06, 3.35; I^2: 0%), however, the reduction was not significant for sedentary behavior (SMD = −0.95; 95% CI: −2.06, 0.15; I^2: 98%). Masini and their colleagues also reported similar findings as they reported that Active-break interventions had a significant effect in increasing PA levels, MVPA and step count among primary school children and their meta-analysis demonstrated a statistically significant result for the step count ($p < 0.00001$, CI 95% −0.71,1.21) (random model I 2 = 0%) [21]. It should be noted that both reviews [21,25], included five matching primary studies and the methodological quality of most of the included studies in the review of [25], was generally low and the risk of bias was very high. Furthermore, five RCTs in the review conducted by Masini and colleagues were poor quality, and 11 cohort studies were medium quality.

Theory-based interventions: One review [24], investigated universal interventions (n=178), 62 interventions were theory based and the duration on average was 44 weeks (Range: 1–410 weeks). 146 studies assessed the PA outcomes as 74 studies used accelerometers, 25 studies used pedometers, 58 used questionnaires and 8 studies used direct observation. 141 included studies in this review evaluated the effectiveness of the intervention on PA outcomes post-intervention, and they recorded at least one significant intervention effect on measured PA, while 48 included studies did not show any significant increase in PA. In terms of long-term effectiveness, nine out of nineteen studies showed at least one significant intervention effect on measured PA, while ten studies did not indicate any intervention effect on the PA. In brief, the review of [24] found a high percentage of effective interventions, some single-feature interventions were found to be effective while no specific feature combination seemed to be associated with better intervention effectiveness. Therefore, theory-based single as well as multi-feature interventions seem to have the potential to improve effectiveness concerning PA, CRF, and sedentary behaviour [24]. It should be noted that the quality of the included studies in this review was unknown since the authors did not assess the risk of bias.

Multi-component interventions: in the review of [26] most of their interventions (70%) were multi-component interventions and they found that interventions in children resulted in a small positive treatment effect in the intervention group compared to the control group (2.14; 95% CI = 0.77, 3.50). There was no significant effect on sedentary behaviors, energy intake, and fruit and vegetable intake but significant reductions were found between groups in BMI kg/m2 (−0.39; 95% CI = −0.47, −0.30) and BMI z-score (−0.05; 95% CI = −0.08, −0.02) in favour of the intervention. However, the quality of most of the included studies in this review was at low risk of bias.

Physical education lessons: The last review of this age group [22] was to examine interventions to increase MVPA content in physical education. Their intervention "Born to

Move physical activity and fitness intervention alongside a regular PE lesson" found favorable intervention effects on children's MVPA in all the studies, with a pooled effect of 14.3% higher lesson time in MVPA in the intervention groups, equivalent to around a 9-minute improvement in MVPA per 1 hour of PE lesson time. The range was from a 4% to 30% difference in the MVPA content of PE lessons. Evidence quality was generally high in this review.

Interventions for Adolescents aged 12 to 18 Years

Out of the twenty-three reviews, only three reviews [18,19,27] investigated interventions conducted in secondary schools for adolescents aged between 12 and 18 years.

Multi-component interventions: Buru and their colleagues reviewed three types of interventions: physical activity, sedentary behavior and nutrition. PA was used in 11 out of 13 reviewed studies, the shortest intervention was four months and the longest was two years with a follow-up of three years. They found that only two of the included studies reported a significant increase in active transport, in terms of change in PA. Furthermore, only four of the included studies reported a significant decrease in BMI, whereas eight studies did not find any significant changes in BMI. Furthermore, three included studies recorded a significant decrease in body fat and only one study reported a significant decrease in body weight for the intervention group as compared to the control group. Therefore, Buru and their colleagues thought that despite the weak evidence of intervention efficacy for most of the reviewed studies, school-based interventions with multi-component combinations of physical activity, nutrition, and alignment to a theory yielded promising results. The quality assessment of included studies revealed that 11 studies were of medium quality and two studies were of high quality.

In the systematic review that was focused on only adolescent girls [19], most of their interventions were multi-component, four interventions were modified PE lessons and two

were educational based, the shortest intervention was six months and the longest was three years. 13 interventions were delivered by the school staff, two were delivered by instructors and two by research team. Ten included studies used accelerometers to measure PA, nine studies used self-report questionnaires and two studies combined self-report and accelerometers. Owen with their colleagues found that school-based interventions provided a small, but significant positive effect on PA levels among adolescent girls ($k = 16$, $g = 0.07$, $p = 0.05$). The quality assessment for included studies revealed that ten studies were classified as having a moderate risk of bias, six studies were weak, three were strong and one had a very strong risk of bias.

Active breaks intervention: The systematic review conducted by [27], aimed to examine the impact of school-based physical activity interventions of "active breaks" on PA levels and other outcomes such as classroom behaviour, cognitive functions and well-being. They reviewed three studies about physically active lessons and active break interventions. Intervention periods varied from 11 weeks to 7 months. They found that two of the included studies demonstrated a positive effect of active breaks on students' classroom behavior and quality of life whereas, one study reported a positive effect in the increase in school physical activity levels, and this effect was not found in the overall levels of PA or the reduction of sedentary behavior. The quality assessment revealed that one was good, one was poor and one was fair quality.

Interventions for Both Children and Adolescents aged 6 to 18 Years

Out of the twenty-three reviews, thirteen reviews [15–17, 28–37] considered interventions conducted in both primary and secondary schools for children and adolescents aged between 6 and 18 years.

Active transport: three reviews [29,32,34] investigated interventions concerning "active school transport" and "active commuting to and from school". In reviews of [29,34], accelerometers were used to measure PA in all included studies. Larouche et al reported that 13 interventions resulted in a statistically significant increase in active school transport whereas eight reported no changes at all, four included studies recorded inconsistent results and five did not record inferential statistics. Campos- Garzón et al included 14 studies of which 11 were cross-sectional and two longitudinal studies, and their meta-analysis found that the overall active commuting to and from school weighted light physical activity (ACS) was 19.55 min (95% CI: 3.84-35.26; $I2 = 99.9\%$, $p < 0.001$) and 68.74 min (95% CI: 6.09-131.39; $z = 2.15$, $p = 0.030$) during the home-school and school-home trips, respectively. For MVPA, the overall ACS weighted MVPA was 8.98 min (95% CI: 5.33–12.62; $I2 = 99.95\%$, $p < 0.001$) during the home-school trip and 20.07 min (95% CI: 13.62-26.53; $I2 = 99.62\%$, $p < 0.001$) during the school-home trip. Schönbach et al included nine studies that investigated seven unique interventions and they reported that only one included study reported positive intervention effects on total MVPA ($\beta = 21.6$ [CI95: 8.7, 34.6]), MVPA from cycling ($\beta = 23.0$ [CI95: 10.7, 35.4]) and MVPA before/after school ($\beta = 12.8$ [CI95: 8.5, 17.2]).

It should be noted that most of the included studies in the reviews of [29,32] were rated as poor quality and had severe limitations in the design and implementation of interventions, therefore, they attributed a low grade for the overall quality of evidence. For the review conducted by [34] most studies had a low risk of bias and medium quality.

Multicomponent interventions: in two reviews [16,17] most of their included interventions were concerning diet, education, and PA. The review of [17] was in Latin America and the review of [16] was in the Middle East and Arabic-speaking countries. [17] reviewed 16 interventions most of which targeted diet alone and two focused on PA compared a curriculum

of PA programming with a conventional PE class, the duration ranged from four months to two years with a minimum follow-up of five months. They reported that above 60% of the included studies recorded a significant effect on at least one dietary/PA or obesity-related outcome, and five included studies reported a significant effect on physical condition tests and/or PA time. While [16] reviewed 17 interventions most of them were multi-component (lifestyle, diet, education) and few studies focused on PA. The duration ranged from six weeks to three years with a maximum follow-up of one year. They reported that eleven included studies recorded statistically significant improvements in the levels of PA among their participants based largely on self-reported outcomes. They also reported that the studies with a follow-up period greater than three months reported sustained PA levels. It should be noted that most of the included studies (13 out of 16) in the review conducted [17] were weak quality. In the review of [16], 14 out of 17 studies had a low risk of bias.

Single component interventions: in the review conducted by [28], they aimed to examine the possibility of school-based interventions for promoting PA and PF and preventing obesity. They reviewed 19 studies and most of the interventions were focused on physical activity-related interventions including expanding the duration of PE or altering its content, performing extracurricular physical activities, participating in activities during breaks and lunch breaks, or providing activity breaks in classes other than PE. They recorded that when the BMI variable was examined in studies more focused on PA, the success rate was 72.72% in eight out of 11 studies included and the success rate was found in only two included studies used PA as support. These findings refer to that PA-oriented interventions are more likely to be successful in the BMI variable. Furthermore, when the PA variable was examined, they found that PA levels of school children significantly increased in a proportion of the studies, and this success rate was achieved in studies focused on orientation and training. This result might show the significance of applying physical activity strategies as the focus of the intervention program to

increase the levels of PA [28]. It should be noted that 10 included studies were rated as strong quality whereas nine studies were moderate quality.

Educational interventions: the review and meta-analysis conducted by [30] aimed to evaluate the effects of educational interventions on promoting regular PA. They reviewed 14 studies and 8 of them were performed as a controlled clinical trial. They reported that the overall estimate of the mean difference demonstrated that interventions had a statistically significant effect on weight loss, the intervention group had a weight loss of 1.02 kg (95% CI: 0.22–4.79 kg) compared with the control group and the effectiveness in included studies that used combined intervention for a longer period of time was higher than one-dimensional interventions. The quality assessment revealed that six included studies were high quality, four studies were medium and four were weak quality [30].

Active desks: the review of [33], investigated the effects of active desks on sedentary behavior, PA, academic achievements, and overall health. They included 23 studies and active desks were in three forms: upright active desk, cycling desk and stability ball. One included study out of 23 found a significant difference in BMI for the interventional group compared to the control group after two years of intervention (−5.24 for BMI percentile) and other studies did not report any change. For sedentary behavior, two included studies noticed that when children used upright active desks spent significantly less time sedentary rather than children in the control group and six included studies reported a significant decrease in sitting time during school hours in the intervention group. Regarding PA, included studies recorded several different outcomes such as light PA, MVPA, step counts, stepping, standing, and walking time. For MVPA, two studies reported contradictory results and one study observed a significant increase in MVPA between pre- and post-intervention. Furthermore, statistically significant increases were reported for standing time in nine included studies. For step counts, one study recorded an increase but without statistical analyses. For stepping time, one study reported an

increase in light PA and MVPA with an objective measurement. Regarding academic performance, only two studies that used upright active desks recorded an increase in the intervention group without any change in concentration. The overall quality of the included studies in the review of [33] was low.

Motivational interventions: The review of [31] examined motivational interventions based on PA as the precursor of psychosocial benefits among children and adolescents. 45 studies were included, and 39 studies presented a quasi-experimental design and between five and twenty weeks were the average durations of the intervention programs. Vaquero-Solís reported that 23 studies out of 45 demonstrated psychological effects, 10 included studies demonstrated psychosocial effects after the intervention and the rest studies reported changes that were not significant. Moreover, the majority of reviewed studies were based on the theoretical models: Achievement Goal Theory and Self-Determination Theory. Therefore, their review demonstrated the significance of motivational processes for the implementation of PA and sport as a precursor of psychosocial changes highlighting the significance of strategies and the duration of interventions/programmes - ie the longer interventions also have a longer lasting impact on behaviour to maintain significant changes over time. The majority of included studies in review of [31] were rated as high quality with few being medium quality.

Geographically specific reviews

The review of [15] examined the effectiveness of interventions to promote PA among children and adolescents in Asian countries. They reviewed 30 studies, 21 included studies were school-based, and 16 studies were cluster RCT. Interventions ranged in duration from one day to 60 months (median 4.5 months). Their primary outcome was changed in PA and PA was assessed by device-based tools such as an accelerometer and pedometer in four studies, and the rest used self-reported such as questionnaires. 17 included studies out of 30 used a theoretical

framework/ model and eight studies used multiple intervention strategies including a combination of PA sessions, exercise, physical activities such as indoor-outdoor PA, aerobic dance, rope skipping activities and PE sessions. The other included studies used educational interventions. 15 studies showed significant increases in PA behaviour or PF and these studies were of moderate-to-high quality. Their evidence also showed that single or multicomponent interventions for short-term (up to six months) including PA sessions, PE, and health education might increase overall PA in Asian children and adolescents.

Rural and urban settings: the systematic review and meta-analysis of [36] aimed to evaluate and compare the effect of rural and urban/suburban school-based PA interventions on total PA among youth. They included 33 studies, and the most common intervention component was PE as used 20 times and classroom-based interventions were used 16 times. In rural studies, the meta-analysis indicated that no statistical significance pooled intervention effect on total PA. In subgroup analysis, they found that the magnitude of the effect was consistently small. The test of group differences was significant only when considering studies separated by design (RCT vs. non-RCT), Qb (38) = 4.97, $p = 0.03$, indicating a significant difference in effect size between interventions with an RCT design and interventions with a non-RCT design. Therefore, in brief, their findings demonstrated that the pooled effect of all school-based interventions to increase total PA was statistically significant but small. When they analysed urban and suburban studies separately, the effect was also statistically significant but small. When they analysed rural studies separately, findings demonstrated a null effect of school-based interventions to increase total PA. It should be noted that the quality of included studies is unknown since [36] did not assess the risk of bias for included studies.

Impact on MVPA and fitness: The review of [37], investigated intervention effectiveness to increase MVPA and improve fitness. There were 89 included studies in the review representing 66,752 individuals. They reviewed 40 multi-component interventions, 19 school time PA, 15

enhanced PE, and 14 before and after school programs. The duration of interventions varied greatly from a minimum of 12 weeks to 6 years, with 10 studies reporting intervention periods of 3 years or longer. Their review showed that interventions based in schools may result in little to no increase in time engaged in MVPA (mean difference 0.73 minutes/d, 95% confidence interval 0.16 to 1.30, little to no decrease in sedentary time (MD -3.78 minutes/d, 95% CI -7.80 to 0.24, may improve PF recorded as maximal oxygen uptake (VO2max) (MD 1.19 mL/kg/min, 95% CI 0.57 to 1.82 and may result in a minimal decrease in BMI z-scores (MD -0.06, 95% CI -0.09 to -0.02. The overall quality of the included studies in the review of [37] was as follows: 33 studies were moderate quality, and 56 studies were low quality.

Impact on HRQoL: The meta-analysis conducted by [35], aimed to assess the effects of PA interventions on several domains of health-related quality of life (HRQoL) and to examine the effectiveness of interventions on the same domains. They reviewed 17 different interventions and most of them were adding extra (PE) lessons to the regular school curriculum with a few of them being short active breaks. Their meta-analysis indicated that pooled effect size (95% CI) estimates for the effect of PA on HRQoL were as follows: 0.179 (0.045, 0.002) for total HRQoL score, 0.192 (0.077, 0.306) for physical well-being, 0.158 (0.080, 0.237) for psychological well-being. In brief, their results conclude that physical exercise improved overall HRQoL and several HRQoL domains, such as physical well-being, psychological well-being, autonomy and parent relation, and social support and peers. However, their findings indicate no positive effect of the exercise programs on the school environment domain. The risk of bias for the included studies in the review of [35] showed that six out of 17 were at high risk of bias, four studies were medium, and seven studies were at low risk of bias.

Quality Assessment of Included Reviews

The methodological quality of the included reviews was classified into four categories: high, moderate, low, or critically low overall quality. Seven reviews were of high quality, four reviews were of moderate quality, five reviews were of low quality, and seven reviews were critically low. Most reviews did not report on the sources of funding for the studies included, except one review [37]. Also, most studies did not provide a list of excluded studies nor justify the exclusions, except for two reviews [16, 23]. Three out of the twenty-three reviews failed to assess the risk of bias in the included studies, and two of them did not consider the risk of bias in individual studies when interpreting/discussing the results [20, 24, 36]. Furthermore, seven studies did not explain their selection of study designs for inclusion in their reviews [16–19, 28, 29, 31]. Nine studies omitted the components of PICO in their research questions and inclusion criteria [17–20, 25, 26, 28, 33, 35], and the authors of seven studies did not perform duplication in study selection or data extraction [16, 17, 19, 20, 22, 31, 36].

Eight out of the twenty-three reviews conducted a meta-analysis. Four of these studies used partially appropriate methods for the statistical combination of results and assessed the potential impact of the risk of bias in the included studies on the results and evidence synthesis [19, 22, 34, 36]. The findings of the quality assessment are presented in Supplemental Table 3 (S3 Appendix in S1 File).

Discussion

The main purpose of this umbrella review was to comprehensively evaluate the effectiveness of school-based physical activity interventions among children and young people aged 6 to 18 years internationally. A total of 23 reviews were included, with seven reviews implemented in primary schools among children aged 6 to 11 years, three reviews implemented in secondary schools among adolescents aged 12 to 18 years, and thirteen reviews targeted both

children and adolescents aged 6 to 18 years. Our ability to come to solid conclusions was limited because the quality of the systematic reviews or meta-analyses was low, or because, as mentioned in several reviews, the quality of the included studies was very weak. Nevertheless, a few approaches seem more hopeful in encouraging more physical activity than others.

This umbrella review identified various school-based interventions, presenting mixed findings, with effectiveness appearing to vary by age. Among the reviews focusing on interventions in primary schools for children aged 6 to 12 years, two interventions—the Daily Mile Programme [20, 23] and Active Break intervention [25]—were highlighted as promising for increasing PA, demonstrating significant improvements in physical activity, physical fitness, and MVPA. The Daily Mile Programme, implemented in over 15,600 schools worldwide, has shown rapid adoption over the last decade [23], with evidence indicating that long-term participation can enhance MVPA by nearly ten minutes a day and positively impact physical fitness. However, children participating in The Daily Mile programme do not maintain MVPA intensity throughout the entire 15 minutes, though any increase in MVPA is beneficial for cardiometabolic health [38]. Nevertheless, to accurately assess the sustainability and full impact of The Daily Mile, higher-quality research with proper randomisation and extended follow-up periods is recommended [23].

Active break interventions were performed in three forms: active breaks, curriculum-focused active breaks, and physically active lessons. Two reviews recorded a significant increase in PA and MVPA with no significant reduction in sedentary behaviour [21, 25]. Therefore, these two specific interventions (TDM and Active breaks), seem to contribute to achieving the public health recommendation of 60 minutes of MVPA per day for children.

Among the reviews that investigated interventions conducted in secondary schools for adolescents aged 12 to 18 years. Multicomponent interventions in two reviews [18,19]

demonstrated a very small and nonsignificant effect on PA levels. These findings are in line with the previous umbrella review in the same field [9] as they reported that school-based interventions targeting adolescents do not have significant effects and are of questionable value. Owen et al suggest that during adolescence, complex physiological and psychological changes occur, which may complicate efforts to change behavior [19].

Reviews including both children and adolescents found mixed findings for school-based interventions. Active transport interventions demonstrated a statistically significant increase in active school transport when PA was objectively measured [29]. For MVPA, the overall ACS weighted MVPA was 8.98 min (95% CI: 5.33–12.62; I2 = 99.95%, $p < 0.001$) during the home-school trip and 20.07 min (95% CI: 13.62-26.53; I2 = 99.62%, $p < 0.001$) during the school-home trip (Campos- Garzón et al., 2023). Therefore, active transport interventions may contribute 50% of the physical activity recommendations in school aged children on school days when both trip directions were actively performed.

Single component interventions with a focus on PA-related interventions including expanding the duration of PE or altering its content, performing extracurricular physical activities, participating in activities during breaks and lunch breaks, or providing activity breaks in classes other than PE demonstrated a small but significant increase in PA levels especially when they focused on orientation and training [28]. Educational interventions that concerning diet, PA and education showed a statistically significant effect on weight loss and a significant increase in PA especially when physical activity programs employed a multi-component approach that integrated into the school curriculum, and included teachers, parents, and students [30]. These results are in line with previous reviews indicating that programs that combine diet and PA produced greater benefits than single strategies alone [39–41]. Furthermore, we found that a long-term intervention was another key component of effective interventions. The results of this review are consistent with those of previous studies that found

that school-based interventions became successful when they had a duration of one school year or more and less successful in those that lasted less than six months [6].

Active desk interventions showed a small effect on BMI reduction, a significant decrease in sitting time during school hours, and statistically significant increases in standing time [33]. Motivational interventions showed psychosocial effects after the intervention when they were based on theoretical models such as achievement goal theory and self-determination theory [31]. These results in line with the previous narrative review [6] examined the effectiveness of school-based interventions in Europe and indicated that ineffective school-based interventions were not based on a well-defined theoretical framework. This indicates the importance of theoretical models in designing school-based PA interventions.

Multi-component interventions demonstrated little to no increase in MVPA, little to no decrease in sedentary time, a minimal decrease in BMI and little improve in PF [37]. Finally, adding extra (PE) lessons to the regular school curriculum with a few of them being short active breaks showed positive effect on several domains of health-related quality of life such as physical well-being and psychological well-being [35]. Nevertheless, schools remain an important setting for promoting PA, since they interact more frequently with school-aged children during the first two decades of their lives than any other institution. Furthermore, as indicated by [42] youth's ability to increase their level of physical activity and sustain it may be heavily influenced by their social environment and their social support system. It is unlikely that someone will begin to walk to school if no one walks to school [42]. Additionally, [31] reported in their review that the behaviour of peers has a profound effect on young people’s behaviour.

Both the methods and reporting of systematic reviews in the topic area could be improved. For example, most reviews failed to report on the sources of funding for the studies included

in the review. Also, most studies failed to provide a list of excluded studies and justify the exclusions. Three reviews out of 23 reviews failed to assess the risk of bias in included studies and two of them did not account for the risk of bias in individual studies when interpreting/ discussing the results [20, 24, 36]. Nine studies did not include the components of PICO in the research questions and inclusion criteria. Despite this, it was difficult to draw concrete conclusions in light of the low quality of some included studies. Consequently, further high-quality research is needed to determine the effectiveness of physical activity interventions.

Strengths and Limitations of Primary Studies Included in Reviews

The key strength of primary studies included in reviews is the range and diversity of programmes evaluated and the range of different outcome measures. In addition, eleven reviews included high to medium-quality primary studies. However, there were some limitations: firstly, the quality of primary studies in some reviews cited as a limitation by review authors. Further, some authors indicated that the primary studies included had a high risk of bias and were unclear in some cases [17, 25, 29, 32, 33, 35, 37]. So, it is impossible to fully trust the conclusions of a systematic review if the primary studies were flawed. Secondly, related to the trade-off between evaluating "real world" programmes and the lack of rigorous RCTs - and the impossibility of blinding which means that depending on self-reported outcomes was problematic.

Strengths and Limitations of the Umbrella Review

The key strength of this umbrella review is that it is the first to systematically assess the most recent scientific evidence on school-based physical activity interventions to promote physical activity among children and adolescents around the world. Furthermore, an in-depth quality assessment was conducted using the latest version of the AMSTAR 2, a tool for assessing systematic reviews. In addition, bringing these findings together has provided a comprehensive picture of the school-based physical activity interventions that are most likely

to increase school children's and adolescents' levels of physical activity. However, this umbrella systematic review has some limitations. Firstly, the searches were limited to studies published in English and Arabic language which may led to missing potentially relevant studies. Secondly, with consideration of the heterogeneity in primary studies designs in each review, different types of interventions, outcomes, duration and measures restricted to the potential synthesis of the findings from different reviews and to directly compare the effectiveness of different intervention types.

Implications of the Findings for Policy Makers and Schools

The findings of this umbrella review have shown that some interventions were promising in increasing physical activity levels in school children and adolescents such as the Daily Mile programme, active breaks, active desks, and multi-component interventions. Other interventions such as multi-component interventions concerned with PA, nutrition and education shown a potential to increase PA and decrease BMI. While effective programmes have been identified, their applicability may vary globally. Thus, future research should aim to enhance the quality of evidence and broaden the implementation context. Moreover, there is a need for more studies with robust designs, like rigorous randomised controlled trials, that have adequate sample sizes, proper control groups, sufficient follow-up periods extending well beyond the intervention, and that employ validated measures of PA and assess sustainability issues.

Conclusion

This umbrella review evaluated the literature on the effectiveness of school-based physical activity interventions for children and young people aged 6 to 18 years internationally. The Daily Mile, Active Break, and Active Transport interventions showed promise in enhancing PA levels, while multi-component interventions were effective in reducing BMI. Long-term interventions, supported by rigorous theoretical and methodological frameworks, also appeared

to increase PA levels. However, the overall conclusions are tempered by the low quality of some studies, highlighting the need for further high-quality research to ascertain the true effectiveness of these interventions.

FUNDING

This review is apart from PhD study funded by the Saudi Arabian Cultural Bureau in London and administered through the doctoral programme run at the University of Sheffield, School of Medicine and Population Health.

CONFLICTS OF INTEREST STATEMENT

No conflict of interest was reported by the authors.

Supporting information

PRISMA Checklist.

Appendix 1. Search strategy.

Appendix 2. Table 2: Characteristics of included reviews.

Appendix 3. Table 3: Quality assessment by AMSTAR version 2

Appendix 4. Studies were excluded during full-text screening with reasons.

References

1. WHO. Childhood Overweight and Obesity. Available online: http://www.who.int/dietphysicalactivity/ childhood/en/ (accessed on 8 December 2023)

2. Hoffman EL, VonWald T, Hansen K. The metabolic syndrome. South Dakota Medicine. 2015 Jan 2.

3. Moore JB, Davis CL, Baxter SD, Lewis RD, Yin Z. Physical activity, metabolic syndrome, and overweight in rural youth. The Journal of rural health. 2008 Mar;24(2):136-42.

4. Inchley JC, Currie DB, Jewell J, Breda J, Barnekow V. Adolescent obesity and related behaviours: trends and inequalities in the WHO European Region, 2002–2014.

5. Buttitta M, Iliescu C, Rousseau A, Guerrien A. Quality of life in overweight and obese children and adolescents: a literature review. Quality of life research. 2014 May;23:1117-39.

6. Drouka A, Brikou D, Causeret C, Al Ali Al Malla N, Sibalo S, Ávila C, Alcat G, Kapetanakou AE, Gurviez P, Fellah-Dehiri N, Masson M. Effectiveness of school-based interventions in Europe for promoting healthy lifestyle behaviors in children. Children. 2023 Oct 11;10(10):1676.

7. Weihrauch-Blüher S, Wiegand S. Risk factors and implications of childhood obesity. Current obesity reports. 2018 Dec;7:254-9.

8. Pyle, S.A., Sharkey, J., Yetter, G., Felix, E., Furlong, M.J. and Poston, W.C., 2006. Fighting an epidemic: the role of schools in reducing childhood obesity. *Psychology in the Schools*, *43*(3), pp.361-376.

9. Mannocci A, D'Egidio V, Backhaus I, Federici A, Sinopoli A, Ramirez Varela A, Villari P, La Torre G. Are there effective interventions to increase physical activity in children and young people? An umbrella review. International Journal of Environmental Research and Public Health. 2020 May;17(10):3528.

10. Lambrinou CP, Androutsos O, Karaglani E, Cardon G, Huys N, Wikström K, Kivelä J, Ko W, Karuranga E, Tsochev K, Iotova V. Effective strategies for childhood obesity prevention via school based, family involved interventions: a critical review for the development of the Feel4Diabetes-study school based component. BMC endocrine disorders. 2020 May;20:1-20.

11. Brown T, Moore TH, Hooper L, Gao Y, Zayegh A, Ijaz S, Elwenspoek M, Foxen SC, Magee L, O'Malley C, Waters E. Interventions for preventing obesity in children. Cochrane Database of Systematic Reviews. 1996 Sep 1;2019(7).

12. Fairclough S, Stratton G. 'Physical education makes you fit and healthy'. Physical education's contribution to young people's physical activity levels. Health education research. 2005 Feb 1;20(1):14-23.

13. Moher D, Shamseer L, Clarke M, Ghersi D, Liberati A, Petticrew M, Shekelle P, Stewart LA, Prisma-P Group. Preferred reporting items for systematic review and meta-analysis protocols (PRISMA-P) 2015 statement. Systematic reviews. 2015 Dec;4:1-9.

14. Shea BJ, Reeves BC, Wells G, Thuku M, Hamel C, Moran J, Moher D, Tugwell P, Welch V, Kristjansson E, Henry DA. AMSTAR 2: a critical appraisal tool for systematic reviews that include randomised or non-randomised studies of healthcare interventions, or both. bmj. 2017 Sep 21;358.

15. Ahmed KR, Uddin R, Kolbe-Alexander TL, Khan A. The effectiveness of physical activity interventions in Asian children and adolescents: a systematic review. Public Health. 2021 May 1;194:48-59.

16. Alalawi A, Blank L, Goyder E. School-based physical activity interventions among children and adolescents in the Middle East and Arabic speaking countries: A systematic review. Plos one. 2023 Jul 3;18(7):e0288135.

17. Chavez RC, Nam EW. School-based obesity prevention interventions in Latin America: A systematic review. Revista de saude publica. 2020 Nov 2;54:110.

18. Buru K, Emeto TI, Malau-Aduli AE, Malau-Aduli BS. The efficacy of school-based interventions in preventing adolescent obesity in Australia. InHealthcare 2020 Nov 25 (Vol. 8, No. 4, p. 514). MDPI.

19. Owen MB, Curry WB, Kerner C, Newson L, Fairclough SJ. The effectiveness of school-based physical activity interventions for adolescent girls: A systematic review and meta-analysis. Preventive medicine. 2017 Dec 1;105:237-49.

20. Hanna L, Burns C, O'Neill C, Coughlan E. A Systematic Review of the Implementation and Effectiveness of 'The Daily Mile'on Markers of Children's Health. International Journal of Environmental Research and Public Health. 2023 Jun 22;20(13):6203.

21. Masini A, Marini S, Gori D, Leoni E, Rochira A, Dallolio L. Evaluation of school-based interventions of active breaks in primary schools: A systematic review and meta-analysis. Journal of science and medicine in sport. 2020 Apr 1;23(4):377-84.

22. Wong LS, Gibson AM, Farooq A, Reilly JJ. Interventions to increase moderate- to- vigorous physical activity in elementary school physical education lessons: Systematic review. Journal of School Health. 2021 Oct;91(10):836-45.

23. Breslin G, Hillyard M, Brick N, Shannon S, McKay-Redmond B, McConnell B. A systematic review of the effect of The Daily Mile™ on children's physical activity, physical health, mental health, wellbeing, academic performance and cognitive function. PLoS One. 2023 Jan 12;18(1):e0277375.

24. Brandes B, Busse H, Sell L, Christianson L, Brandes M. A scoping review on characteristics of school-based interventions to promote physical activity and cardiorespiratory fitness among 6-to 10-year-old children. Preventive Medicine. 2022 Feb 1;155:106920.

25. Carrasco-Uribarren A, Ortega-Martínez A, Amor-Barbosa M, Cadellans-Arróniz A, Cabanillas-Barea S, Bagur-Calafat MC. Improvement of in-school physical activity with active school-based interventions to interrupt prolonged sitting: a systematic review and meta-analysis. International Journal of Environmental Research and Public Health. 2023 Jan 16;20(2):1636.

26. Nally S, Carlin A, Blackburn NE, Baird JS, Salmon J, Murphy MH, Gallagher AM. The effectiveness of school-based interventions on obesity-related behaviours in primary school children: a systematic review and meta-analysis of randomised controlled trials. Children. 2021 Jun 8;8(6):489.

27. Masini A, Ceciliani A, Dallolio L, Gori D, Marini S. Evaluation of feasibility, effectiveness, and sustainability of school-based physical activity "active break" interventions in pre-adolescent and adolescent students: A systematic review. Canadian Journal of Public Health. 2022 Oct;113(5):713-25.

28. Yuksel HS, Şahin FN, Maksimovic N, Drid P, Bianco A. School-based intervention programs for preventing obesity and promoting physical activity and fitness: A systematic review. International journal of environmental research and public health. 2020 Jan;17(1):347.

29. Larouche R, Mammen G, Rowe DA, Faulkner G. Effectiveness of active school transport interventions: a systematic review and update. BMC public health. 2018 Dec;18:1-8.

30. Moeini B, Rezapur-Shahkolai F, Bashirian S, Doosti-Irani A, Afshari M, Geravandi A. Effect of interventions based on regular physical activity on weight management in adolescents: a systematic review and a meta-analysis. Systematic Reviews. 2021 Dec;10(1):1-3.

31. Vaquero-Solís M, Iglesias Gallego D, Tapia-Serrano MÁ, Pulido JJ, Sánchez-Miguel PA. School-based physical activity interventions in children and adolescents: A systematic review. International journal of environmental research and public health. 2020 Feb;17(3):999.

32. Schönbach DM, Altenburg TM, Marques A, Chinapaw MJ, Demetriou Y. Strategies and effects of school-based interventions to promote active school transportation by bicycle among children and adolescents: a systematic review. International journal of behavioral nutrition and physical activity. 2020 Dec;17(1):1-7.

33. Guirado T, Chambonnière C, Chaput JP, Metz L, Thivel D, Duclos M. Effects of classroom active desks on children and adolescents' physical activity, sedentary behavior, academic achievements and overall health: a systematic review. International journal of environmental research and public health. 2021 Mar 10;18(6):2828.

34. Campos- Garzón P, Sevil- Serrano J, García- Hermoso A, Chillón P, Barranco- Ruiz Y. Contribution of active commuting to and from school to device- measured physical activity levels in young people: A systematic review and meta- analysis. Scandinavian Journal of Medicine & Science in Sports. 2023 Nov;33(11):2110-24.

35. Bermejo-Cantarero A, Sánchez-López M, Álvarez-Bueno C, Redondo-Tébar A, García-Hermoso A, Martínez-Vizcaino V. Are physical activity interventions effective in improving health-related quality of life in children and adolescents? A systematic review and meta-analysis. Sports health. 2023 Aug 22:19417381231190885.

36. Pfledderer CD, Burns RD, Byun W, Carson RL, Welk GJ, Brusseau TA. School- based physical activity interventions in rural and urban/suburban communities: A systematic review and meta- analysis. Obesity Reviews. 2021 Sep;22(9):e13265.

37. Neil-Sztramko SE, Caldwell H, Dobbins M. School- based physical activity programs for promoting physical activity and fitness in children and adolescents aged 6 to 18. Cochrane Database of Systematic Reviews. 2021(9).

38. Ekelund U, Luan JA, Sherar LB, Esliger DW, Griew P, Cooper A, International Children's Accelerometry Database (ICAD) Collaborators. Moderate to vigorous physical activity and sedentary time and cardiometabolic risk factors in children and adolescents. Jama. 2012 Feb 15;307(7):704-12.

39. Oosterhoff M, Joore M, Ferreira I. The effects of school- based lifestyle interventions on body mass index and blood pressure: a multivariate multilevel meta- analysis of randomized controlled trials. Obesity reviews. 2016 Nov;17(11):1131-53.

40. Williams AJ, Henley WE, Williams CA, Hurst AJ, Logan S, Wyatt KM. Systematic review and meta-analysis of the association between childhood overweight and obesity and primary school diet and physical activity policies. International Journal of Behavioral nutrition and Physical activity. 2013 Dec;10(1):1-22.

41. Wolfenden L, Nathan NK, Sutherland R, Yoong SL, Hodder RK, Wyse RJ, Delaney T, Grady A, Fielding A, Tzelepis F, Clinton- McHarg T. Strategies for enhancing the implementation of school- based policies or practices targeting risk factors for chronic disease. Cochrane database of systematic reviews. 2017(11).

42. McIntosh JR, Jay S, Hadden N, Whittaker PJ. Do E-health interventions improve physical activity in young people: a systematic review. Public Health. 2017 Jul 1;148:140-8.

Click here to access/download
Supporting Information
Supporting information.docx

Click here to access/download
Supporting Information
PRISMA 2020 cheklist.docx
