# Supplementary material for: Umbrella review of international evidence for the effectiveness of school-based physical activity interventions
Source: PLoS One. 2024 Jun 13;19(6):e0304513. doi: 10.1371/journal.pone.0304513 (PMC11175402; doi:10.1371/journal.pone.0304513)
Supplement: Attachment — Submitted filename: Response to Reviewers.docx [file pone.0304513.s004.docx]

**Reviewer #1 Comments:**

Reviewer #1: Title
1. Very long title, try to summarize the title

Changed to “**Umbrella Review of International Evidence for the Effectiveness of School-based Physical Activity Interventions”**

**Reviewer #1:** Keywords
2. The words "systematic review", "school-based", "physical activity" and "interventions" are already included in the title, here they should be replaced by synonyms.

Changed to "evidence synthesis, "physical activity programmes" and " health promotion".

**Introduction**
3. In the introduction you mention in particular obesity and sedentary lifestyle, and talk briefly about physical inactivity, I believe it would be important to reinforce in the introduction with quantitative data the size (percentage) of this physical inactivity among this world population. I believe it is important since all the interventions studied certainly aimed to reduce sedentary behavior and/or increase the level of physical activity and consequently remove this population from the "physically inactive" classification.

Additional text and relevant references have been added to this section:

“The World Health Organization (WHO) identifies childhood obesity as a significant public health concern in the 21st century. In 2016, a staggering 41 million children below the age of five were reported to be overweight, predominantly residing in developing nations [1]. This issue is closely linked to sedentary behaviors, which have become increasingly prevalent; currently, it is estimated that up to 81% of adolescents worldwide are not meeting the recommended levels of 60 minutes of moderate to vigorous physical activity (MVPA) daily [2]. Such physical inactivity is a major contributor to the rise in non-communicable diseases, such as diabetes, cardiovascular diseases, and metabolic syndrome in later life [3,4].

The adverse effects of childhood obesity extend beyond physical health, impacting psychological aspects as well; children facing obesity issues often suffer from poor self-image and low self-esteem, elevating their risk of developing eating disorders and leading to a diminished quality of life [5]. Contributing to the complexity of this challenge are significant societal and lifestyle changes observed over recent decades in Europe, characterized by a shift towards high consumption of unhealthy fats and added sugars and a marked decrease in physical activities [6,7].”

4. About PRISMA item 3 "Describe the rationale for the review in the context of existing knowledge" it needs to be improved.

As suggested we have revised and improved the clarity of this section (see pages 4 and 5).

"Numerous programmes have been developed to promote healthy lifestyles and combat childhood obesity, with schools commonly the setting for such interventions. This strategic choice leverages the school’s capacity to reach children across diverse ethnic and socio-economic backgrounds, ensuring that health-promoting efforts are broadly accessible [8,9]. Schools not only provide a structured environment for implementing physical activity programmes but also inherently promote physical activity through compulsory sports education and encouraging active commuting among students [10–12]. Despite these widespread initiatives, there remains a significant variation in the success rates of these programmes, often due to differences in programme design, implementation fidelity, cultural adaptations, and other contextual factors.

A previous umbrella systematic review [9] synthesized evidence on interventions to increase physical activity among children and young people across various settings, including school-based programmes. It found that interventions involving physical activity

embedded within the school curriculum, long-term initiatives, initiatives with teacher involvement, and family support show the most promise in enhancing physical activity levels. The review includes studies conducted in school, preschool, family, and community settings, highlighting the comprehensive scope of its analysis and the pivotal role of schools in promoting physical activity.

This umbrella systematic review aims to bridge an evidence gap by updating and providing a comprehensive overview that includes more recent studies on the effectiveness of school-based physical activity interventions to promote physical activity among children, and young people aged 6 to 18 years old worldwide.”

**Methods**
5. Inclusion and Exclusion Criteria: The exclusion criteria serves to exclude studies that were included according to my inclusion criteria, but which, due to some specific characteristic, do not meet the other inclusion criteria. So when you say that the inclusion criterion is "Interventions that were implemented in schools and quantitative evaluations of PA" you already make it clear that " Reviews included interventions not based on schools" will not be included, so I suggest rethinking all the exclusion criteria, as most of the exclusion criteria seem to me to already be included in the inclusion criteria.

The table was deleted and the text changed to clarify the inclusion criteria as follows:

***Selection Criteria***

This umbrella review included systematic reviews and meta-analyses that incorporated various study designs, including randomised controlled trials (RCTs), cluster-randomised controlled trials (c-RCT), controlled trials (CT), quasi-experimental studies, and observational studies. Excluded from consideration were studies that did not adopt a systematic review approach, narrative reviews, and primary studies. The reviews were deemed eligible if they involved participants between the ages of 6 to 18 years. Included studies evaluated at least one intervention for increasing physical activity among the targeted population, were delivered in school and included quantitative evaluations of PA. Reviews published before 2018 were excluded because the previous related umbrella review was based on a search for studies published from January 2010 until November 2017 [9]. Studies published in Arabic or English language were included.

6. The reference [9] that you mention as if your study were a "continuation" used the following databases PubMed, Scopus and the Cochrane Library. So I would like to know what the justification is for using CINAHL and not the Cochrane Library? Considering the characteristics of the databases, I believe that the Cochrane Library would be more appropriate. Another detail is that the reference you cited used 3 languages (English, Italian and Spanish) and you only used two (Arabic and English) I believe it would be important to consider at least one more language.

Thank you for highlighting this issue on our database selection which we have now addressed in explanatory text on the choice of databases.

“We chose CINAHL over the Cochrane Library because it provides extensive coverage of public health and educational literature, essential for understanding school-based interventions. CINAHL's broad inclusion of peer-reviewed articles and proceedings makes it particularly suited to our research focus. While the Cochrane Library is valuable, especially in clinical settings, it offers less in public health education and school-level interventions.” We also appreciate the concern that the limitations of our database search strategy and restriction to English and Arabic language reviews may have led to our searches not being exhaustive but were constrained by resource considerations as this review was undertaken by the first author within a PhD programme. We have mitigated these limitations in several ways including forward and backward (citation and reference) searching and checking the reference lists of other related systematic reviews. These strategies provide reassurance that it is unlikely that there is a substantive number of additional studies which would meet our inclusion criteria and change our overall conclusions about the nature of current evidence in this field. We have highlighted this limitation further in the Discussion section, in the section on the strengths and limitations of our review.

**Results**
7. Appendix 2. Table 2: Characteristics of the included reviews. In the first column it would be very important to include in which country the studies were developed, so that you could also summarize by country in the item "Review characteristics" and not just by continent as stated.

Thank you for your suggestion to include the country of origin for each study listed in Appendix 2, Table 2 of our review. We appreciate the value this detail could add to understanding the contextual influences on the effectiveness of school-based physical activity interventions.

However, implementing this change presents significant challenges. Our umbrella review includes 23 systematic reviews, which collectively include 691 primary studies. The scope and volume of these studies make it logistically difficult to accurately track and report each study’s country of origin within the confines of our current review format.

Given the extensive nature of the data and our resource constraints, we categorised the reviews by continent to provide a manageable and still informative geographical overview. This approach was intended to balance detail with clarity, allowing us to discuss regional trends without becoming overly granular, which could potentially obscure the broader patterns relevant to our findings.

We understand the importance of geographical specificity and will consider ways to incorporate more detailed location data in future updates or related studies where feasible. For now, we have ensured that our categorisation by continent provides a meaningful insight into the variability and applicability of the interventions across different global contexts and highlighted that this is a limitation of the umbrella rveiw.

8. About Appendix 2. Table 2: Characteristics of the included reviews. It would be very important for it to appear in the body of the text and not just as an appendix, but for that to happen it should be "summarized" as it is very extensive, I believe that some "column" information could be excluded without losing the essence.

Thank you for your recommendation to include the contents of Table 2 from Appendix 2 directly within the body of the text for enhanced accessibility. We appreciate the intent behind this suggestion to make key data more immediately available to readers.

We carefully considered this possibility during the manuscript preparation. However, due to the extensive nature of the table, which includes detailed characteristics of 23 reviews and 691 primary studies, incorporating it in full within the main text proved impractical. We found that such inclusion would significantly disrupt the flow and readability of the manuscript, making it less accessible to readers.

To ensure that all pertinent information is still readily accessible and highlighted appropriately, we have synthesised and integrated the critical data from Table 2 throughout the manuscript, particularly in the results and discussion sections. This approach allows us to maintain a narrative that is both informative and digestible, without the overwhelming detail that a full table would impose within the text.

We believe this method effectively balances detailed reporting with narrative clarity, adhering to the journal's standards for comprehensive yet reader-friendly presentations.

We hope this clarifies our rationale and ensures the manuscript meets the expectations of both the review panel and the broader readership. Thank you again for your comments.

9. In this excerpt "Quality Assessment of Included Reviews The methodological quality of the included reviews was classified into four categories: high, moderate, low, or critically low overall quality. Seven reviews were of high quality, four reviews were of moderate quality, five reviews were of low quality, and seven reviews were critically low." It is important to include the references of the 7 studies that have high quality, the 4 moderate quality and so on, this information must be verified throughout the results section.

We have added this information on page 20. “The methodological quality of the included reviews was classified into four categories: high, moderate, low, or critically low overall quality. Seven reviews were of high quality [4,5,14–16, 22,23], four reviews were of moderate quality [17–20], five reviews were of low quality [6, 8, 9, 12, 21], and seven reviews were critically low [1–3, 7, 10, 11, 13].”

**Conclusion**
10. better explain the contribution of this review to future studies

**We have added some additional text to the Conclusion (page 26)**

“This umbrella review evaluated the literature on the effectiveness of school-based physical activity interventions for children and young people aged 6 to 18 years internationally. The Daily Mile, Active Break, and Active Transport interventions showed promise in enhancing PA levels, while multi-component interventions were effective in reducing BMI. Long-term interventions, supported by rigorous theoretical and methodological frameworks, also appeared to increase PA levels.

“The findings from this review are crucial for shaping future research directions, emphasising the need for enhanced methodological quality and more robust RCTs. These should include adequate sample sizes, appropriate control groups, and extended follow-up periods to accurately assess the sustainability and true effectiveness of interventions. Moreover, the review underscores the importance of integrating validated physical activity measures into future studies, alongside an evaluation of sustainability issues.”

**Reviewer #2: Methods**

Why didn’t you check out Web of Science?

Thank you for your comment regarding the selection of databases for our systematic review of reviews. We carefully considered which databases to include based on their coverage of literature pertinent to our research topic. Our decision to search PubMed, Scopus, and CINAHL was guided by their extensive coverage of medical, scientific, and public health literature, which are central to our review's focus on school-based physical activity interventions.

While Web of Science is a comprehensive and valuable resource, our preliminary search indicated that the key literature in our field was adequately covered by the selected databases. Specifically, Scopus provides a broad and overlapping coverage with the Web of Science, especially for the scientific and health-related articles that are crucial for our study. Furthermore, given the specific scope of our review, CINAHL was essential for its specialised coverage of public health literature, which the Web of Science does not uniquely offer.

We ensured that our search strategy was comprehensive and capable of capturing the relevant studies necessary to answer our research questions, without duplication that might not add value to our analysis. However, we appreciate your point and will consider including Web of Science in future updates of our review to further verify the comprehensiveness of our literature search.

Write how many of these studies had done quality assessment?

Abstract (Results)

**Added to Abstract (results section)**

20 out of 23 reviews assessed the quality of primary studies.

**Thank you so much for your valuable suggestions.**
